# Trend analysis of COVID-19 mis/disinformation narratives–A 3-year study

**DOI:** 10.1371/journal.pone.0291423

**Published:** 2023-11-17

**Authors:** Bonka Kotseva, Irene Vianini, Nikolaos Nikolaidis, Nicolò Faggiani, Kristina Potapova, Caroline Gasparro, Yaniv Steiner, Jessica Scornavacche, Guillaume Jacquet, Vlad Dragu, Leonida della Rocca, Stefano Bucci, Aldo Podavini, Marco Verile, Charles Macmillan, Jens P. Linge

**Affiliations:** 1 Piksel S.r.l., Ispra, VA, Italy; 2 Athens University of Economics and Business, Athens, Greece; 3 Engineering S.p.A., Rome, RM, Italy; 4 FINCONS, Corso Magenta 56, Milano, MI, Italy; 5 Gi Group S.p.A., Varese, VA, Italy; 6 European Commission, Joint Research Centre, Unit T.5, Ispra, VA, Italy; La Trobe University - Melbourne Campus: La Trobe University, AUSTRALIA

## Abstract

To tackle the COVID-19 infodemic, we analysed 58,625 articles from 460 unverified sources, that is, sources that were indicated by fact checkers and other mis/disinformation experts as frequently spreading mis/disinformation, covering the period from 1 January 2020 to 31 December 2022. Our aim was to identify the main narratives of COVID-19 mis/disinformation, develop a codebook, automate the process of narrative classification by training an automatic classifier, and analyse the spread of narratives over time and across countries. Articles were retrieved with a customised version of the Europe Media Monitor (EMM) processing chain providing a stream of text items. Machine translation was employed to automatically translate non-English text to English and clustering was carried out to group similar articles. A multi-level codebook of COVID-19 mis/disinformation narratives was developed following an inductive approach; a transformer-based model was developed to classify all text items according to the codebook. Using the transformer-based model, we identified 12 supernarratives that evolved over the three years studied. The analysis shows that there are often real events behind mis/disinformation trends, which unverified sources misrepresent or take out of context. We established a process that allows for near real-time monitoring of COVID-19 mis/disinformation. This experience will be useful to analyse mis/disinformation about other topics, such as climate change, migration, and geopolitical developments.

## Introduction

Soon after the initial spread of the the SARS-CoV-2 virus, the World Health Organisation (WHO) coined the term “infodemic” to describe the social phenomenon of having “too much information including false or misleading information in digital and physical environments during a disease outbreak” [1]. Indeed, the coronavirus pandemic evidenced how information disorder, which tends to increase in contexts of uncertainty, emergency, and general confusion, can have tangible, harmful consequences. A review of 31 studies on health mis/disinformation found that mis/disinformation as well as incorrect interpretations of health information have an adverse effect on society as they increase vaccine hesitancy, have a negative impact on people’s health, including their mental health, and delay the provision of healthcare [2]. Concerned by the potential damage that false or misleading information could cause at a time of crisis, academia and international institutions developed policy guidelines to counteract the spread of mis/disinformation during the pandemic. Scholars highlighted the crucial role played by social media, providing recommendations to mitigate the spread of mis/disinformation on digital platforms [3–5]. At the same time, the European Commission well acknowledged the challenge of tackling disinformation [6] and continues to improve its policy framework by working with a broadening range of stakeholders [7, 8].

Researchers investigated the propagation of COVID-19 related mis/disinformation from a plethora of angles. Ecker and colleagues investigated the psychological, social, and affective drivers leading people to believe and endorse COVID-19 mis/disinformation as well as individual reluctance to accept corrected information [9]. Leon and colleagues identified the main characteristics of mis/disinformation and developed a typology to classify several types of hoaxes [10]. Clemente-Suarez and colleagues searched specific COVID-19 related keywords on prominent academic databases to provide a critical narrative review, stressing how mis/disinformation can diminish individuals’ trust in vaccines and reinforce scepticism about the severity of the pandemic [11]. Similarly, Agley and Xiao qualitatively investigated the impact of COVID-19 related conspiracy theories on individuals, considering several key socio-demographic factors [12]. Their academic endeavour relied on previous research identifying the top five most prominent narratives in April 2020 [13]. Focusing specifically on Twitter, Darius and Urquhart investigated how specific conspiracy theories proliferated among communities before and during the first lockdown in the United Kingdom, adopting a hybrid approach combining quantitative and qualitative methods [14]. Furthermore, Siwakoti and colleagues carried out an extensive cross-regional analysis of COVID-19 related mis/disinformation narratives by collecting 5,613 unique stories from over 80 countries in 35 languages from the beginning of the pandemic until December 2020 [15]. Their approach underlined the importance of monitoring the development of specific mis/disinformation trends in relation to specific events as well as phases of the COVID-19 pandemic. Of particular interest for this manuscript is the development of a codebook with narratives, sources, and distribution channels for each identified story [15].

While drawing from the existing literature, our approach distinguishes itself for the large quantity of the data analysed, the high number of sources included in the investigation, and the extended time period considered. We collected over 1.3 million COVID-19 related news articles from unverified sources–“news sites” that had been indicated by independent fact checkers and other experts as frequently spreading mis/disinformation–from 36 countries and 24 languages in the period between the 1 of January 2020 and the 31 of December 2022. Our objective was to develop an ad-hoc codebook detailing the main mis/disinformation narratives around COVID-19. This approach aligns with current research on misleading narratives around COVID-19 [11, 14, 15]. The identification of these narratives aims at supporting the development of effective public health messaging campaigns, as research shows that narrative-based messaging tends to yield more effective outcomes than fact-based messaging [16, 17]. By identifying which mis/disinformation narratives are prevalent in a given period of time or in a given geographic location, public health communicators are empowered to tackle mis/disinformation through targeted messaging.

## Materials & methods

Our methodology was based on our internal surveillance briefing production workflow, which is similar to the approach used by Coan and colleagues to measure climate change denial trends across time [18]. Modern NLP approaches have been extensively applied to COVID-19 research [19] for several application areas, including mis/disinformation detection.

Our process consisted of (a) creating a list of unverified sources and a keyword filter to gather news articles on a weekly basis, (b) carrying out an article extraction from our database of unverified sources, translating non-English articles into English, and applying clustering algorithms to the articles in order to group articles about the same topic, (c) using human annotators for both the creation of our hierarchical labelling (referred to as the codebook) and subsequent labelling of individual articles, (d) using the data to train a BERT based classifier that gradually replaced the human annotation, (d) carrying out limited human supervision in order to limit the impacts of data drift.

Unlike most COVID-19 misinformation detection research utilising similar NLP models, that used either a four label annotation (Fake, Biased, Irrelevant, Other) [20], or a three label entailment task (Agree, Disagree, No stance) along with a bank of misconception claims [21], we intended to fully annotate each narrative that we deemed important to flag in a hierarchical fashion (Supernarrative, Narrative, Subnarrative) and build a model able to track the evolution of such narratives across time.

For (a) we put together a list of unverified sources, that is, “news sites” that had been indicated by fact checkers and other independent experts as frequently spreading mis/disinformation. We exclusively focused on webpages spreading mis/disinformation without taking into account social media channels. This choice was mainly driven by the type of infrastructure at our disposal for the retrieval of the data. Throughout the monitored period, our database of unverified sources contained 460 unique domains, covering 35 countries and 24 languages.

We then constructed a query containing several coronavirus-related keywords in the 24 official EU languages, Arabic, Chinese, Hebrew, Japanese, Korean, and Russian, which was constantly updated to cover new relevant keywords, such as newly discovered variant names (see query in S1 File, version of December 2022). Similar approaches, have used either Boolean queries [22] or hashtag filter in social media [23].

For (b), we carried out a multilingual keyword extraction from our database of unverified sources in order to collect articles about COVID-19. We then translated non-English text to English using an in-house translation tool and, due the existence of similar or even identical articles across the news landscape, we applied two types of clustering algorithms to facilitate a more diverse training dataset. We used our in-house Hierarchical Cluster Analysis (HCA) implementation on both the TF-IDF matrix and the matrix based on Latent Semantic Analysis (LSA) [18, 19] and picked the centroid of those clusters for further annotation, similar to [24, 25].

For (c), two annotators carried out the annotation following an inductive approach: throughout the coding process, they identified the main mis/disinformation narratives present in the dataset and built a codebook used to annotate all the articles of the dataset on a weekly basis.

Although previous studies focusing on the spread of narratives were reviewed [4, 11, 13–16], for the purpose of this investigation, we defined a narrative as a “particular way of explaining” an event, as per the definition of the word “narrative” in the Cambridge dictionary [26].

Throughout the annotation process, we adopted a multi-level hierarchical structure of narratives, ranging from “supernarratives” to “narratives” to “subnarratives.” In order to do that, we carried out a systematic content analysis, identifying the main COVID-19 mis/disinformation narratives and organising them into different levels based on their level of specificity. On the top level, we collected rather general “supernarratives”. On the second level, we identified “narratives” presenting different angles within each supernarrative. On the third level, we pinpointed “subnarratives” containing more detailed stories within each narrative. For some narratives, the annotators agreed that two levels were sufficient. For example, the annotators agreed that under the supernarrative “criticism of restrictions,” it was sufficient to distinguish the articles into different narratives based on whether they criticised a specific measure implemented during the pandemic or whether they criticised the restrictions in general, without needing to go into further detail on the “subnarrative” level. Therefore, this supernarrative breaks down into narratives, such as “anti-lockdown narratives,” “anti-mask narratives,” “criticism of restrictions in general,” but the narratives do no further break down into subnarratives. In other cases, narratives were organised according to a more detailed, hierarchical structure. In the case of articles about vaccination, for example, the annotators decided that they would assign them to the supernarrative “vaccine-related narratives.” During the annotation process, they noted that unverified sources were covering the COVID-19 vaccination process from several angles. Many articles claimed that the vaccines were ineffective or that they caused side effects or that they were deadly, etc.; this observation led the annotators to create the narrative “anti-vax narratives” and the subnarratives “vaccines are ineffective,” “vaccines cause side effects,” “vaccines are deadly,” etc.

While the two annotators carried out the annotation independently, they built a unified codebook, comparing codes and revising the codebook based on the appearance of new narratives and the disappearance of old ones. When one of the annotators had doubts over the annotation of a single item, the two annotators carried out the annotation of the item in question jointly.

We carried out steps (a), (b), and (c) on a daily and then weekly basis. In the meantime, we aggregated all the data and built a unified codebook. The final codebook comprises 12 supernarratives, 51 narratives, and 44 subnarratives. After the codebook was finalised, the annotators re-annotated all the items tagged with an outdated narrative according to the revised codebook (see S2 File).

For (d), we developed an automatic narrative classifier, based on a multi-lingual pre-trained BERT model [27]. BERT-based models have been used before to monitor and detect misinformation both in the context of COVID-19 [20] and to conduct trend analysis of climate change denial claims over time [17]. Our classifier was built on our codebook of COVID-19 mis/disinformation narratives and the pre-trained model was fine-tuned with a subset of 30,000 annotated items. A training-validation-testing split was created. The results of the evaluation on the test set can be found below (see Table 1). For detailed results of the classifier, please see S1 Table.

**Table 1:** Evaluation results. Results of the Precision, Recall and F1 evaluation of BERT classifier on test set.

F1-micro (Accuracy)	F1-macro	Precision	Recall
0.84	0.80	0.81	0.80

For (e), the results were evaluated on a weekly basis to account for the existence of data-drift in the incoming articles. The distribution of the errors was compared with the one from the test set, and where appropriate, the results were sampled and inspected in order to spot the existence of data drift.

Our methodology is subject to four main limitations. First, it is necessary to note that our findings are contingent on the unverified sources monitored throughout the data collection process. S1 Fig illustrates the country coverage of our list of unverified sources, clearly showing that the databased of unverified sources this investigation is based on contains many US, Western European, and Russian sources while it is lacking Latin American, African, and Asian sources. This limitation is contingent on the fact checkers and independent experts that we relied on in order to compile the list of unverified sources, given that they are mostly focused on the US and Europe. This translates into an inability to quantitatively compare narratives across countries. Our country-based analysis is merely meant to highlight the narratives that were most widespread in any given country for which we annotated a significant number of articles. Second, it is important to acknowledge the role that the clustering algorithms mentioned in point (a) may have played on the final set of articles that was annotated, given that they automatically excluded from further examination around 90 percent of the articles extracted from unverified sources. This might have resulted in an over-representation of articles that were republished by multiple sources as opposed to articles published by fewer—but potentially more influential—sources. Third, it is important to acknowledge that, due to the artefacts of the automatic translation process, the clusters tended to be composed mostly of articles of the same language. Finally, although the annotators discussed the codebook at length, it cannot be excluded that annotators’ biases could have affected the annotation process.

## Results

This section summarises the main findings of our analysis of the 58,625 manually annotated COVID-19 related articles from unverified sources.

**Fig 1:**
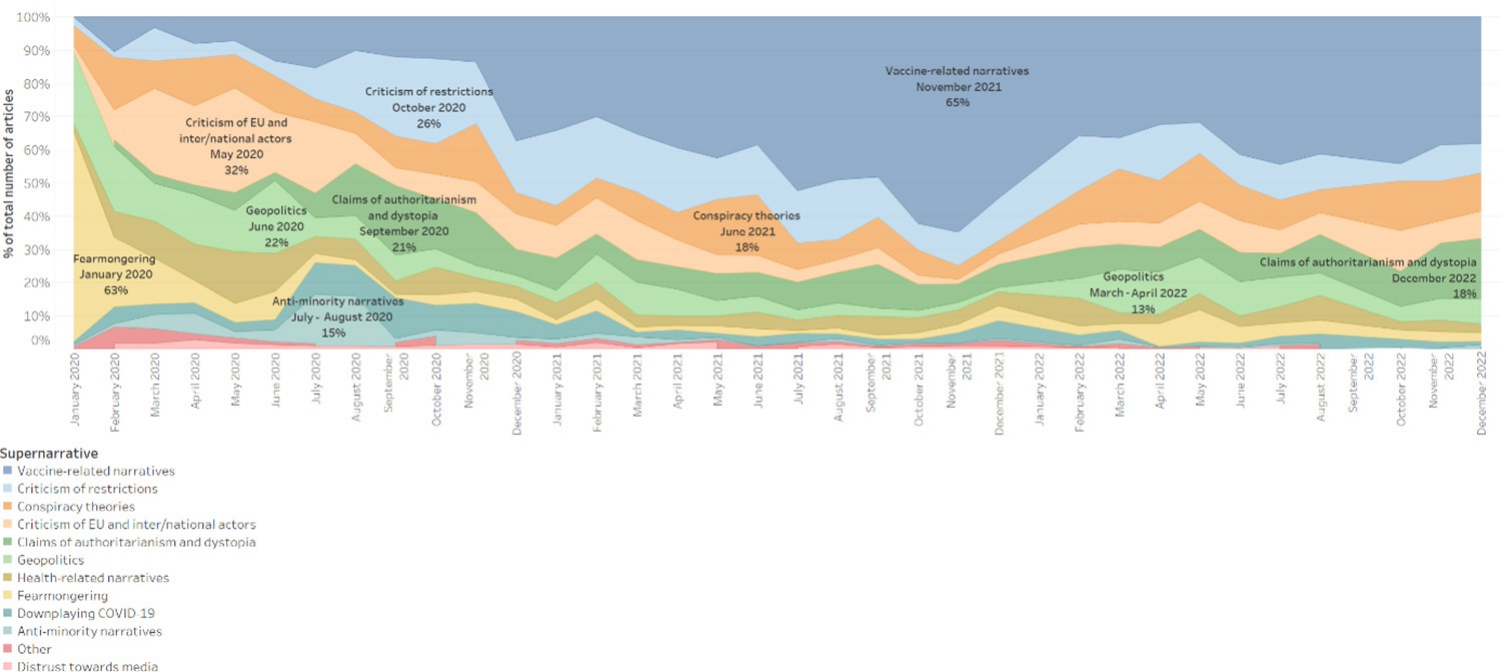
Trend analysis of supernarratives. The stacked time area chart shows the spread over time of the 12 supernarratives (01 January 2020–31 December 2022). The chart allows the reader to identify peaks in the coverage of each supernarrative.

Over the analysed period, we identified 12 supernarratives related to COVID-19 mis/disinformation. Fig 1 shows the spread over time of these 12 supernarratives, highlighting ten significant peaks.

The “**fearmongering**” supernarrative peaked in the first months of the pandemic, when people feared the spread and potential impact of the novel coronavirus, and subsided in spring 2020, as milder temperatures led to a decrease of COVID-19 cases and a lifting of restrictions. During the process of near real-time annotation, many articles were classified as “fearmongering” in the initial stage of the pandemic based on the knowledge available at the time. Taking this into account, articles predicting “up to 4.4 million deaths” published in February 2020 were classified as spreading fearmongering, not knowing that at the time of writing, the global death toll would have surpassed 6.7 million deaths [28].

In January 2020, articles spreading fearmongering represented over 60 per cent of the annotated sample. They reported on the spread of the novel coronavirus, the increasing death toll in China, and the general unpreparedness for a global health crisis. Despite authorities’ appeals to keep calm, unverified sources spread fearmongering claims about the virus, its mutation, and its uncontrolled spread. Similarly, they employed fearmongering language in their reporting of the first restrictions, including many countries’ decision to cancel flights to and from China and major public events. They presented the outbreak in China as an “apocalypse,” where “humanity [was] facing a perfect storm,” while labelling the virus “mysterious” and a “killer [virus].” In February 2020, unverified sources started spreading claims that the virus could reinfect those who had already had COVID-19, and it could be even deadlier the second time around. Many articles reported that the health crisis would lead to food and facemask supply shortages. It is plausible that such articles had an impact on the unmatched level of panic buying that characterised the onset of the pandemic on a global scale [29]. Unverified sources referred to this phenomenon as a “zombie apocalypse.”

Toward the end of March 2020, unverified sources started to heavily engage in “**criticism of [the] EU and inter/national actors**” over their response to the pandemic. They accused governmental institutions and international organisations of mismanaging the pandemic and of lacking international solidarity, especially toward Italy, the first Western country to be hit by the coronavirus. Within the supernarrative “criticism of EU and inter/national actors,” we identified three separate narratives: “criticism of the EU”, “criticism of national governments”, and “criticism of international organisations.”

In May 2020, the supernarrative “criticism of EU and inter/national actors” represented over 30 per cent of the whole dataset. Almost 40 per cent of these items criticised the European Union and its institutions for mismanaging the pandemic, being inactive, refusing to implement border controls to stop the spread of the coronavirus within the EU, and abandoning Italy in times of need. Unverified sources tied the alleged lack of support among Member States to an interview with Hungarian American investor George Soros, who said, “The European Union could break apart in the wake of the new coronavirus pandemic unless the block issued perpetual bonds to help weak members such as Italy”[30]. At the beginning of the emergency, Italian unverified sources often stated that Italy had been “abandoned by the EU”, that the “coronavirus reveal[ed] that Europe [was] dying,” and that Italy was the “sick man of Europe”, calling for an “ItalExit”. Indeed, unverified sources often talked about “ItalExit,” “DeutschExit,” and “CorExit” (exit from the EU of the core Member States), arguing that the pandemic would constitute “the last nail in the coffin of the EU.” Moreover, many articles criticised NextGenerationEU–the EU’s stimulus package to boost the bloc’s recovery—for the uneven distribution of funds among Member States. Many articles accused the EU of having the wrong priorities: of prioritising climate change over the coronavirus emergency or of attempting to use the pandemic to prevent Brexit.

Within the “criticism of international organisations” narrative, in May 2020, nearly 15 per cent of the articles targeted the World Health Organisation (WHO). They criticised WHO’s changing guidelines concerning the health emergency, and they widely reported that US President Donald Trump threated to permanently stop US funding if the organisation did not demonstrate that it was not “a puppet of China.” Other articles criticised the United Nations (UN) in general and the North Atlantic Treaty Organisation (NATO).

The remaining 50 per cent of articles was annotated as “criticism of national governments.” Unverified sources criticised national governments for mismanaging the pandemic—they portrayed lockdowns as a mistake, and they reported inflated death tolls. They also criticised government officials who said in May 2020 that COVID-19 vaccines might be mandatory. Sweden was often the focus of unverified sources: at first, the Scandinavian country was praised for not imposing a lockdown and tight restrictions, but when infections peaked, it was strongly criticised for not providing adequate COVID-19 treatment to home-care patients; it was even reported that health professionals were suspected of euthanising elderly patients.

Following the initial peak in the first half of 2020, the supernarrative “criticism of EU and inter/national actors” decreased to less than five per cent of the annotated data in the summer of 2021 and to only one per cent at the end of 2021. However, in October 2022, unverified sources started to intensify once again their criticism of the EU after the European Medical Agency (EMA) recommended the approval of COVID-19 vaccines for children from 6 months of age [31]. Amid a European Public Prosecutor’s Office (EPPO) investigation into the acquisition of COVID-19 vaccines in the European Union [32], unverified sources also ramped up their criticism of European Commission President Ursula von der Leyen, asking for her resignation.

In June 2020, the supernarrative “**geopolitics**” registered a significant peak, reaching 22 per cent of the annotated data. Articles annotated as related to geopolitics mainly focused on the management of the pandemic, international help to countries in need, and the development and deployment of COVID-19 treatments and vaccines. Within the “geopolitics” supernarrative, we identified "anti-China" and “pro-China narratives,” “anti-Russia” and “pro-Russia narratives”, “anti-US narratives,” “anti-West narratives,” and “other geopolitical narratives.”

Among the “anti-China narratives,” we identified subnarratives accusing the country of being responsible for the pandemic, supplying other countries with faulty medical equipment, and not being trustworthy in its public health communications. While unverified sources were mostly critical of China, their reporting on Russia was mostly positive. While they criticised China for shipping faulty medical equipment to countries in need, they praised Russia for sending medical personnel and equipment to Italy in March 2020. While they questioned China’s official narrative on the origin and spread of the coronavirus, they praised Russia’s management of the pandemic, reporting that containment efforts were successful and ended in Moscow. Unverified sources also praised Russia for the development of new drugs against COVID-19 and of a COVID-19 vaccine–which would come to be known as the Sputnik V vaccine–reporting that it would protect against infection for more than two years, and that Russia would be able to supply the entire world. Regarding Russia’s efforts in vaccine development, they claimed, “While the West is hiding, Russia is working” (see in-depth analysis of the “pro-Russia” subnarrative “vaccine and other drugs” below). Pro-Russia articles often portrayed the West as “russophobic.”

Following the peak in June 2020, the “geopolitics” supernarrative dropped to an average of 5 per cent of the analysed dataset for the following year and a half. However, the supernarrative peaked again at 13 per cent of the annotated dataset in both March and April 2022. Following the beginning of Russia’s military aggression against Ukraine, unverified sources argued that the “conflict between Russia and Ukraine could worsen the global crisis” and claimed that the SARS-CoV-2 virus had been developed in “US biolab facilities in Ukraine.” At the same time, amid the spread of a new COVID-19 wave in China, unverified sources accused the country of mismanaging the pandemic and of using “lockdowns to get people vaccinated.”

The “**criticism of restrictions**” and “**claims of authoritarianism and dystopia**” supernarratives both peaked in the autumn of 2020–47 per cent of the data from September and October 2020 was allocated to these two supernarratives, which portrayed coronavirus restrictions as a means to implement authoritarian regimes and increase surveillance and control over citizens. During the summer of 2020, thanks to warmer temperatures, many countries managed to reopen for tourism, implementing as few measures as possible to keep the virus under control. At the end of the holiday season, however, with the second COVID-19 wave approaching, restrictions were tightened again, and anti-lockdown protests started to unfold.

Within the “criticism of restrictions” supernarrative, nearly 30 per cent of articles spread “anti-lockdown narratives.” Unverified sources portrayed lockdowns as a mistake and even a crime, describing them as the “greatest intrusion on civil liberties” and “health dictatorship.” They argued that not only were lockdowns ineffective at stopping the spread of the virus, but that they were also harmful—they would kill more people than COVID-19 itself. Finally, they reported that many scientists and doctors were against lockdowns, citing several open letters and petitions against the “devastating effects on public health” of lockdowns.

Another 15 per cent of the articles annotated as “criticism of restrictions” spread “anti-mask narratives.” These articles reported on alleged scientific publications “proving” that there was no direct evidence that facemasks prevented viral infection, and argued that mandatory mask-wearing was unconstitutional, and its primary purpose was to foster fear. With the start of the school year in September 2020, unverified sources focused on children, describing the use of masks in schools as pointless, a major threat, harmful to children, and even as child abuse. Some articles leveraged the environmental impact of masks to push their anti-mask agenda. Finally, unverified sources portrayed Sweden as a role model, reporting that it never implemented a lockdown or a mask mandate, and yet it presented the lowest coronavirus death rate in Europe in the autumn of 2020.

In our analysis, we look at the “criticism of restrictions” and “claims of authoritarianism and dystopia” supernarratives together because unverified sources often argued that coronavirus restrictions were aimed at slowly restricting citizens’ liberties and imposing authoritarian, dictatorial regimes.

The “claims of authoritarianism and dystopia” supernarrative represented over 20 per cent of the annotated data from September and October 2020. 80 per cent of these articles were annotated as related to the “turn to authoritarianism” and “dystopia” narratives. These two narratives mainly referred to claims that the “global police state” was swiftly rising, COVID-19 rules were authoritarian and arbitrary, governments were “fascist,” mankind was becoming a “victim of the big brother syndrome,” and constitutional freedom was obsolete. Unverified sources argued that “totalitarianism in practice” was implemented by giving more power to the police and the army, establishing dedicated COVID-19 police forces to control citizens’ compliance with restrictions, using personal data collected through COVID-19 surveillance improperly, and obliging ordinary people to snitch on neighbours who violated coronavirus restrictions. Virtual schooling was presented as dangerous “police state education.”

The remaining 20 per cent of articles within the “claims of authoritarianism and dystopia” supernarrative was annotated as related to the “censorship of dissenting voices” and “censorship by digital platforms” narratives. These articles claimed that individuals or communities who expressed dissenting opinions were censored. Some focused on social media platforms, accusing them of censoring posts that were not aligned with the official COVID-19 narrative.

The “criticism of restrictions” and “claims of authoritarianism and dystopia” supernarratives registered a small peak at the end of 2021 –in December 2021, they constituted 20 per cent of the dataset. This can be explained by the fact that due to the fast spread of the Omicron variant, restrictions were tightened again in December 2021, with anti-lockdown protests ensuing [33].

The “claims of authoritarianism and dystopia” supernarrative peaked again at the end of 2022 –in December 2022, it represented 18 per cent of the annotated articles. Half of these were attributed to the “censorship by digital platforms” narrative. Following Elon Musk’s acquisition of Twitter, unverified sources focused once again on the issue of freedom of expression on social media and the alleged censorship that had taken place on Twitter throughout the coronavirus pandemic.

The “**conspiracy theories**” supernarrative was persistent throughout the analysed period–it constituted an average of 10 per cent of the sample—but it reached nearly 20 per cent in June 2021 and 15 per cent in October 2022. Within the “conspiracy theories” supernarrative, nearly 75 per cent of the articles were annotated as related to the “coronavirus escaped from Wuhan lab” narrative. Unverified sources strongly pushed the argument that the novel coronavirus had been engineered at and leaked from the Wuhan Institute of Virology, and that the Chinese government was involved in a cover-up. They cited a “Wuhan collaborator,” who claimed that his Chinese colleagues had created “killer coronaviruses,” and they circulated a video, which—they claimed—showed bats in cages inside the Wuhan laboratory and “disproved” and “embarrassed” WHO scientists, who would have to admit that they had dismissed the lab-leak theory “due to fear of racism accusations.” Similarly to what is reported above, unverified sources accused Big Tech of censoring the lab-leak theory because it was not aligned with the official narrative. Interestingly, many of the articles annotated as related to the “coronavirus escaped from Wuhan lab” narrative originated from American unverified sources, and attacked top US epidemiologist Dr Anthony Fauci, claiming that he had purposefully ignored a warning that “COVID could have been engineered” and he was acting deceptively.

Throughout the course of the annotation process, the annotators discussed at length whether the “coronavirus escaped from Wuhan lab” narrative should be moved from the “conspiracy theories” supernarrative to the “geopolitics” supernarrative, given that the topic also received attention on mainstream media [34], and the coverage often presented a clear geopolitical dimension. WHO’s inquiry into the origin of the virus, for example, led to an increase of the articles annotated as “geopolitics—anti-China narratives—China cannot be trusted.” Nevertheless, the artificial origin of the virus remains a theory and, at the time of writing, there is no evidence supporting it. It was, therefore, decided to leave the “coronavirus escaped from Wuhan lab” narrative under the “conspiracy theories” supernarrative.

In 2022, some conspiracy theories adapted to the changed geopolitical landscape–unverified sources started to claim that that the SARS-CoV-2 virus had been developed by the US in biolaboratories in Ukraine in order to “create bioagents that can target certain ethnic groups” and commit a “genocide.” They also claimed that “globalists” in the West had instigated Russia’s war against Ukraine “to distract from [the] bombshell [of the SARS-CoV-2 virus being developed in Ukraine.]”

The last supernarrative presenting a clear peak is the “**vaccine-related**” supernarrative, which surged in the autumn of 2020 as preliminary data on the effectiveness of COVID-19 vaccines became available [35]. We annotated over 21,000 articles as related to the “vaccine-related” supernarrative (almost 36 per cent of all annotated data), identifying four main narratives: “anti-vax narratives”, “anti-vax conspiracy theories”, “anti-mandatory vaccination narratives”, and “unconfirmed claims about vaccine development”. The first three narratives are analysed in detail below. The fourth narrative referred to stories about vaccine development across many countries and stakeholders. It represented a relatively small amount of data (less than three per cent of all articles annotated as “vaccine-related”) and it was mainly concentrated in the first few months of the pandemic when the effectiveness of COVID-19 vaccines under development was still uncertain.

Beside the supernarratives elaborated above, we also identified supernarratives related to “**health-related narratives**,” “**downplaying COVID-19**,” “**anti-minority narratives**,” “**distrust towards media**,” and “**other**” narratives.

The “health-related” supernarrative corresponded to 5.5 per cent of the annotated articles. These articles spread false health-related claims about COVID-19, such as claims that nicotine prevented coronavirus infection, and false claims about COVID-19 treatments, such as claims that antimalarial drug Chloroquine and antiparasitic drug Ivermectin were effective against COVID-19. We also identified articles promoting “home remedies” against COVID-19, such as the consumption of yoghurt, whiskey, different types of herbal teas, etc.

The “downplaying COVID-19” supernarrative corresponded to nearly four per cent of the annotated articles. Within the supernarrative, we identified five narratives–that the hype around the pandemic was “hysteria;” that COVID-19 was less dangerous than it was commonly portrayed to be, even less dangerous than the flu; that the spread of the virus and the COVID-19 death toll were overestimated; that COVID-19 tests were not effective at detecting the virus and, therefore, not reliable; and that the pandemic was over or almost over.

The “anti-minority” supernarrative corresponded to less than two per cent of the annotated articles, and it mainly consisted of “anti-immigrant” and “anti-Muslim narratives.” In the summer of 2020, with 15 per cent of the data annotated as “anti-minority” supernarrative in both July and August, unverified sources portrayed immigrants and Muslims as COVID-19 superspreaders and accused them of not complying with restrictions. In this period, Italian unverified sources were especially adamant in spreading “anti-immigrant narratives.” Toward the end of 2021, unverified sources claimed that pharmaceutical companies did not want to get refugees vaccinated because they feared that minorities could sue them following adverse reactions to the vaccines. This storyline seemed to be aimed at depicting refugees as uncollaborative and vaccine manufacturers as fearful of prosecution over the safety of COVID-19 vaccines. In 2022, the main anti-immigrant narratives focused on Ukrainian citizens fleeing the war and seeking refuge in Europe–unverified sources warned their readers that a “wave of unvaccinated Ukrainian refugees” was arriving in Europe and accused the newcomers of rejecting “‘free’ COVID injections.” We also detected narratives against other minorities–antiziganist and antisemitic narratives, and claims accusing the African American community in the US of not following coronavirus restrictions.

The “distrust towards media” supernarrative corresponded to less than one per cent of the annotated articles, and it consisted of attacks against mainstream media and fact checkers. Unverified sources accused mainstream media of being an instrument of propaganda, censoring anti-restriction and anti-vax protests, and leveraging people’s fear to push for “totalitarian control.” A relatively small number of articles focused on fact checkers, accusing them of being an instrument of censorship and profiting from it.

The “other” supernarrative combined narratives that did not fit within the rest of the codebook, but were deemed relevant for our analysis. It constituted one per cent of the annotated articles. It included clickbait stories and sensational reporting, such as stories about armed criminals stealing 600 rolls of toilet paper in Hong Kong, women fighting in supermarkets over toilet roll, and elderly people accidentally burning their money while trying to disinfect bank notes; stories about VIPs who tested positive for COVID-19; and articles focused on criticising top US epidemiologist Dr Anthony Fauci and climate activist Greta Thunberg. While articles criticising Dr Fauci were persistently present over the analysed period, articles criticising Greta Thunberg were predominant in the first half of 2020, and they mainly attacked the climate activist for trying to pull focus away from the pandemic onto the climate crisis.

Details on all supernarratives, narratives, and subnarratives can be found in the codebook, see S2 File.

**Fig 2:**
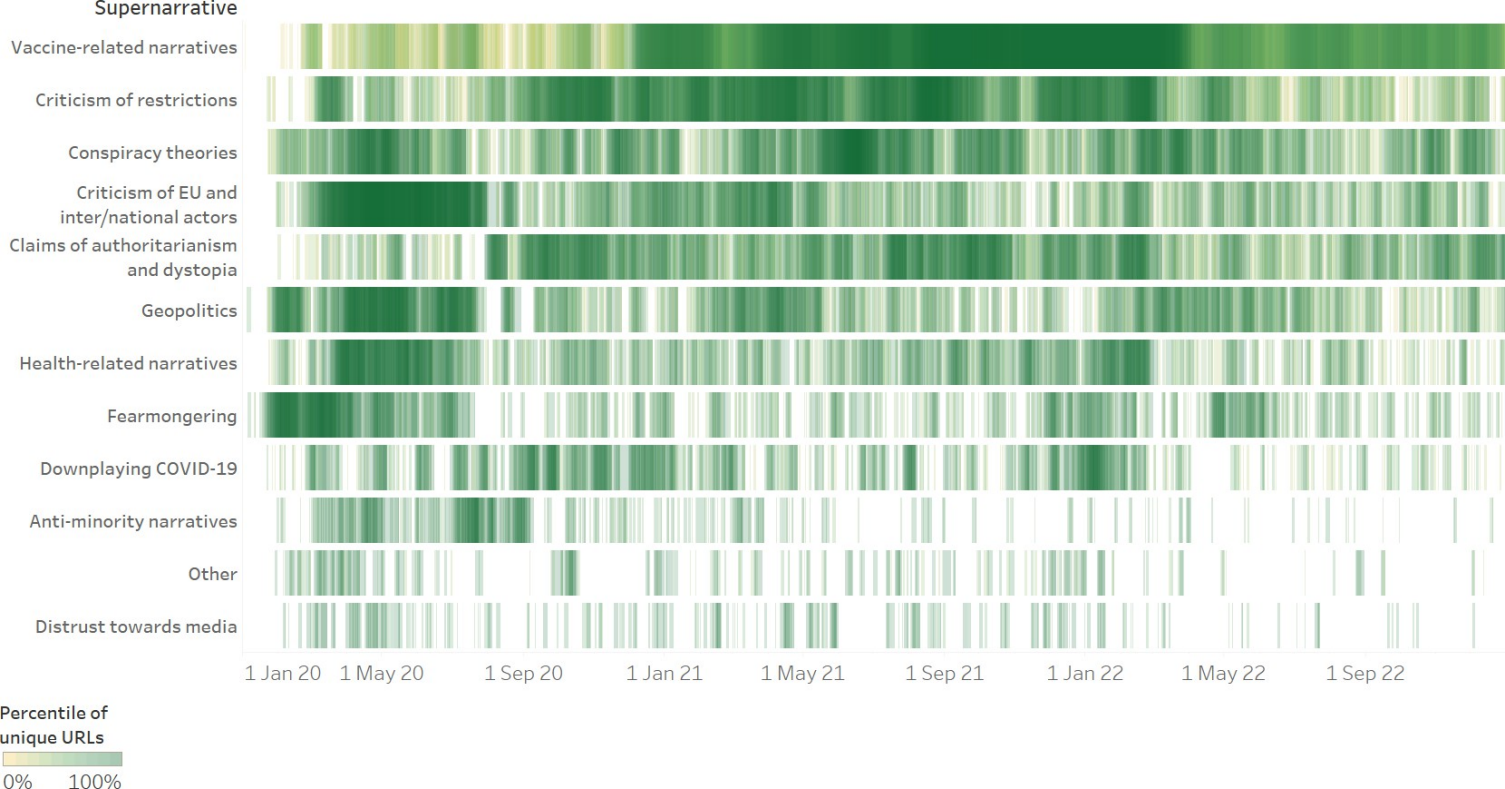
Gantt chart of supernarratives. The Gantt chart shows the spread over time of the 12 supernarratives (01 January 2020–31 December 2022). The chart allows the reader to both analyse the evolution of a given supernarrative overtime and to compare the spread of different supernarratives.

Fig 2 provides a different visualisation of the spread over time of the 12 supernarratives. The Gantt chart clearly shows that whereas the “fearmongering,” “criticism of EU and inter/national actors,” “criticism of restrictions,” and “claims of authoritarianism and dystopia” supernarratives were predominant during the early phase of the pandemic, “vaccine-related narratives” became the main concern of unverified sources in 2021 and 2022.

We identified three types of anti-vax content: “anti-vax narratives” undermining the safety and effectiveness of COVID-19 vaccines and discouraging readers from getting vaccinated; “anti-mandatory vaccination narratives” taking a stance against compulsory vaccination and fomenting fear over its possible implementation; and “anti-vax conspiracy theories” alleging that COVID-19 vaccines were part of a scheme by the elites to further exert their control over the masses.

While “anti-vax conspiracy theories” were present since the onset of the pandemic, “anti-vax narratives” and “anti-mandatory vaccination narratives” started to increase sharply in November 2020 (see additional S2 Fig and additional S3 Fig shows the overall evolution for reference) as preliminary data on the effectiveness of COVID-19 vaccines became available [28].

**Fig 3:**
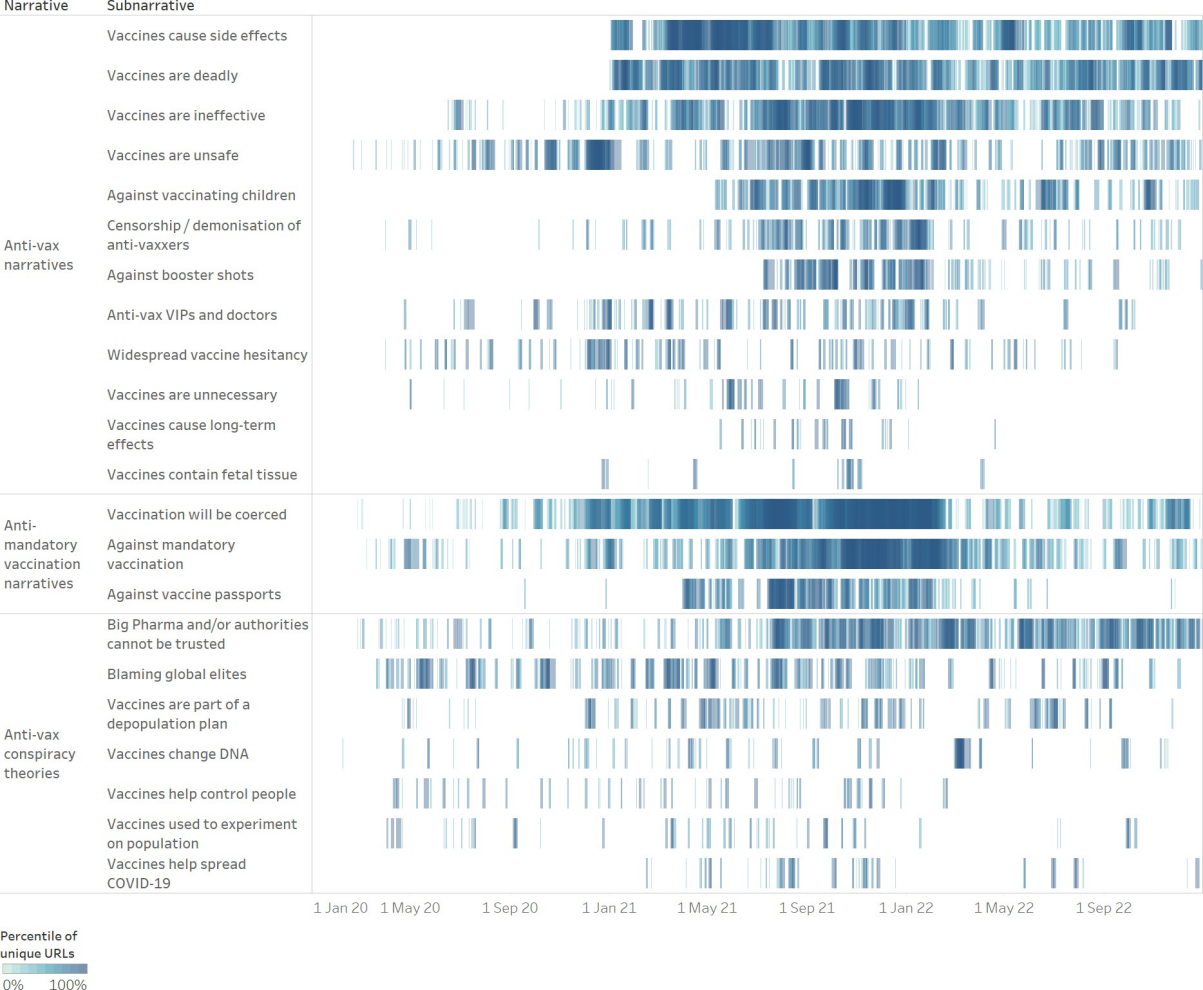
Anti-vax subnarratives. The Gantt chart shows the spread over time of all anti-vax subnarratives, divided by narratives (01 January 2020–31 December 2022). The chart enables the reader to both analyse the evolution of a given subnarrative over time and to compare the spread of different subnarratives.

Among “anti-vax narratives,” (see Fig 3 for an overview of all anti-vax subnarratives) the most widespread subnarratives were “vaccines cause side effects” and “vaccines are deadly.” Both subnarratives were identified at the very beginning of 2021, shortly after the UK [36], the US [37], and the EU [38] had started their COVID-19 vaccination campaigns. Interestingly, the articles often referred to truthful reports of health problems and deaths following vaccination, which were, however, misrepresented or grossly exaggerated. On the one hand, they misleadingly reported on the possible side effects and deaths of vaccine recipients in such a way as to suggest that the vaccines were the cause of injury or death. For example, they often misrepresented data from official pharmacovigilance databases, which, in order to detect possible safety issues, contained all reports of suspected reactions and deaths following vaccination, even if there was no indication of a causal link, claiming that COVID-19 vaccines had killed tens of thousands and injured millions. On the other hand, they put a spotlight on verified reports of vaccine death and injury, in order to make them seem widespread and foment fear. Finally, while unverified sources mainly focused on possible—albeit extremely rare—severe reactions to COVID-19 vaccines, such as blood clots and myocarditis, they also falsely claimed that COVID-19 vaccines caused infertility, miscarriages, cancer, and AIDS.

Another popular subnarrative was “vaccines are ineffective.” It was detected in July 2020, when COVID-19 vaccines were still under development, and it evolved over time. Up until the start of vaccination campaigns, the analysed articles mostly referred to expert statements dwelling on legitimate questions about the effectiveness of vaccines, such as whether vaccines would prevent people from transmitting the virus or whether they would be effective against new strains of coronaviruses, often exaggerated and taken out of context. Once vaccination campaigns were underway, unverified sources mostly shifted to misleadingly report on highly vaccinated countries with high numbers of COVID-19 cases and vaccinated individuals contracting and dying of COVID-19, in order to argue that COVID-19 vaccines were ineffective at preventing infection and death.

Similarly widespread—in terms of overall article count—was the subnarrative “vaccines are unsafe,” which was, however, more evenly distributed over time as it was first identified in February 2020. While strongly tied to the “vaccines cause side effects” and “vaccines are deadly” subnarratives, as they all indicated that COVID-19 vaccines were dangerous, the “vaccines are unsafe” subnarrative based the argument on claims that COVID-19 vaccines contained unsafe ingredients, such as graphene; they led to the creation of new variants; they led to Antibody-Dependent Enhancement (ADE), which allegedly caused vaccinated individuals to have damaged immune systems and be more vulnerable to infection as well as severe COVID-19; and claims that COVID-19 vaccines were developed too quickly and, therefore, were not tested properly.

The “against vaccinating children” subnarrative appeared in mid-May 2021, shortly after the US Food and Drug Administration (FDA) had expanded the Emergency Use Authorisation (EUA) for the Pfizer-BioNTech vaccine to include adolescents 12 through 15 years of age [39]. The analysed articles argued that children should not be vaccinated, as COVID-19 vaccines were dangerous and were not worth the risk, especially for children, who were said to be barely affected by COVID-19.

The “censorship / demonisation of anti-vaxxers” subnarrative was detected in the first quarter of 2020, but its frequency intensified in the second half of 2021, as health passes proving prior infection, vaccination or a recent negative test became a requisite for more and more activities in several countries, and anti-vaxxers became more vocal about their opposition to COVID-19 vaccines and passes. The analysed articles argued that individuals who refused to get vaccinated were censored through authoritarian means or demonised–single-handedly blamed and punished for the failure of the vaccination campaign.

The “against booster shots” subnarrative was identified at the beginning of September 2021, shortly after the US FDA had expanded the emergency use approval for the Pfizer-BioNTech and Moderna vaccines to include a third dose for immunocompromised individuals [39, 40]. The analysed articles argued that booster shots, just like the first and second dose, were unsafe and would be required indefinitely.

The “anti-vax VIPs and doctors” subnarrative was detected in April 2020, but its frequency increased as the public conversation focused more and more on COVID-19 vaccines. It referred to claims by influencers, politicians, and doctors that were either anti-vax or misconstrued as such. A separate subnarrative was created for these claims because of their expected higher impact, given that the reader may have perceived them as coming from an authoritative and/or trusted source.

**Fig 4:**
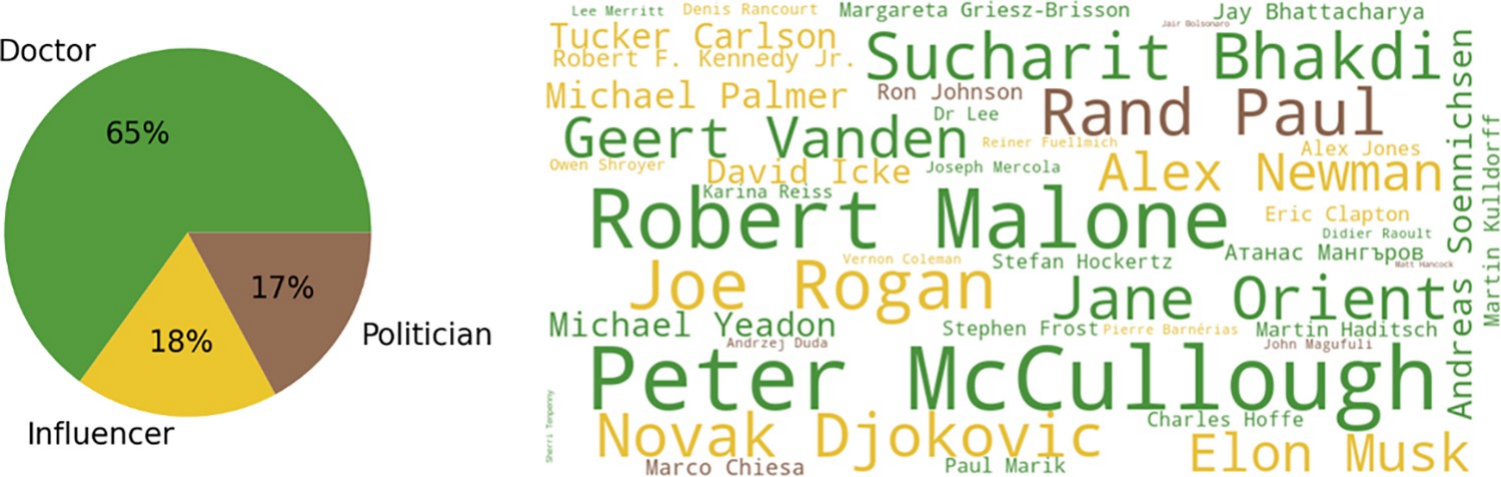
Entity detection. The pie chart shows the distribution of actors in the articles annotated as related to the subnarrative “anti-vax VIPs and doctors.” The word cloud shows the main actors, colour-coded based on the actor category they were assigned to.

Out of a total of 457 articles allocated to the subnarrative “anti-vax VIPs and doctors,” we manually annotated 65 per cent as referring to doctors, 18 per cent as referring to influencers, and 17 per cent as referring to politicians, as shown in Fig 4.

The large number of articles relaying the words of anti-vax doctors shows that doctors are not only important actors for informing the general public about public health measures, but they can also be influential sources of mis/disinformation. The “doctor” category included prominent figures whose medical views have been widely discredited, such as American cardiologist Peter McCullough, whose claims about the dangers of COVID-19 vaccines have been repeatedly debunked by fact checkers [41], and American physician Robert Malone, who *The New York Times* called a “misinformation star” [42]. Among the doctors, we also identified scientists from other disciplines (Biochemistry, Physics, Computer Science, etc.) with no or little experience in human or veterinary medicine. While entity extraction can be used to get a quick overview of the messages related to specific actors, filtering the dataset by language sheds light on country-specific trends, e.g. German unverified sources often cited retired university professor Sucharit Bhakdi while French unverified sources often cited microbiologist Didier Raoult.

Public figures with a large following—such as tennis player Novak Djokovic, entrepreneur and business magnate Elon Musk, and anti-vax propagandist Robert F. Kennedy Jr.—also played an important role in the public debate (see Elon Musk’s tweets predicting “zero new cases” or stating that “kids are essentially immune” in March 2020 [43, 44] or spreading anti-vax memes in December 2021 [45].

The “politician” category included politicians who made false and misleading statements about vaccine safety—such as US Senators Rand Paul [46] and Ron Johnson [47]—as well as other politicians who while not openly anti-vax, were portrayed as such by unverified sources—such as Swiss politician Marco Chiesa and Polish President Andrzej Duda, who made claims against mandatory vaccination [48].

Among the “anti-vax narratives,” the “widespread vaccine hesitancy” subnarrative was rather evenly distributed over the analysed period, but it showed a peak between December 2020 and January 2021 that coincided with the start of COVID-19 vaccination campaigns in the UK, US and EU. The collected articles provided biased reporting on vaccine hesitancy, often focusing on surveys showing that large segments of a country’s population said they would not get vaccinated against COVID-19. These articles appeared to be aimed at questioning the validity of a vaccination programme that would only involve part of the population and would, therefore, not lead to herd immunity.

The “vaccines are unnecessary” subnarrative was present even before the development and approval of COVID-19 vaccines, but it became more widespread throughout the course of 2021. It mainly referred to claims that COVID-19 vaccines were unnecessary or even pointless and a herd immunity approach and/or treatment would be preferable.

The “vaccines cause long-term effects” subnarrative was first picked up in May 2021. While closely related to the “vaccines cause side effects” subnarrative, the “vaccines cause long-term effects” subnarrative mainly referred to claims that the long-term effects of COVID-19 vaccines were unknown, and it could, therefore, not be excluded that COVID-19 vaccines would have negative long-term effects.

Finally, the “vaccines contain fetal tissue” subnarrative, first identified in December 2020, referred to the common anti-vax argument that vaccines, including COVID-19 vaccines, were unethical because they contained aborted fetal cells.

Among “anti-mandatory vaccination narratives,” the most widespread subnarrative was “vaccination will be coerced.” While it was present from the first quarter of the analysed period, it became much more widespread starting from November 2020 when preliminary data on COVID-19 vaccines became available [35]. It generally referred to claims that vaccination would be coerced, either by making it mandatory or by making life impossible for those who refused to get vaccinated until they were forced to change their minds.

The “against mandatory vaccination” subnarrative was predominant in the first half of 2020 when it was still unknown whether the COVID-19 vaccines under development would be safe and effective, but it later subsided. It returned in the second half of 2021 as health passes started to be implemented in several countries. The analysed articles opposed mandatory vaccination on the basis that it infringed upon fundamental rights.

The “against vaccine passports” subnarrative grew in April 2021 after the European Commission unveiled its plan to implement a “Digital Green Certificate,” which would allow EU citizens who had been vaccinated, had tested negative or had recovered from COVID-19 to travel more freely within the EU [49]. The analysed articles argued that vaccine passports should be banned as they infringed upon individual rights and discriminated against unvaccinated individuals. The “against vaccine passports” subnarrative was particularly widespread among Italian unverified sources. This can be explained by the fact that Italy was one of the countries with the widest-ranging applications of the EU Digital COVID Certificate, requiring a “super green pass” showing proof of vaccination or recovery to attend indoor restaurants, sports events, concerts, and theatres, and a basic “green pass,” which could be obtained following a negative COVID-19 test, to use public transport and access places of work [50].

Among “anti-vax conspiracy theories,” the most widespread subnarrative was “Big Pharma and / or authorities cannot be trusted.” It was present throughout the analysed period, and it referred to allegations that Big Pharma and world governments could not be trusted with the development and distribution of vaccines as they had their own dark agendas.

The “blaming global elites” subnarrative, also present throughout the analysed period, referred to allegations that COVID-19 vaccines were the product of a dark scheme by the global elites—spearheaded by billionaire Bill Gates—who were either behind the pandemic or were taking advantage of it to further their vaccination agenda.

Other anti-vax conspiracy theories were “vaccines [make recipients infertile and] are part of a depopulation plan,” mRNA “vaccines change [people’s] DNA,” “vaccines help control people” through the injection of microchips, “vaccines [are] used to experiment on [the] population,” and “vaccines help spread COVID-19” as they facilitate coronavirus infection.

**Fig 5:**
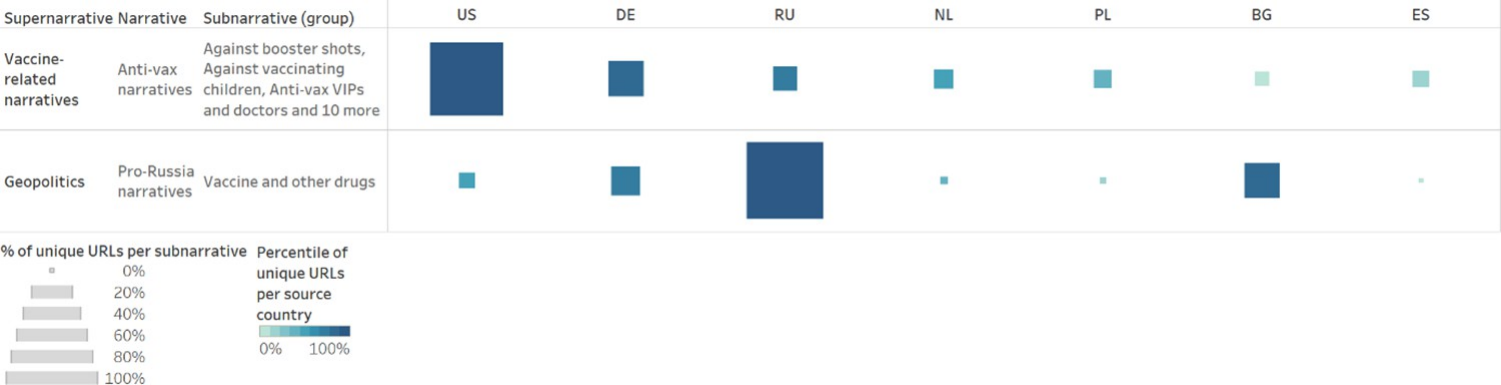
Top country distribution anti-vax/Sputnik V. The table shows the source country distribution of “anti-vax narratives” and “pro-Russia narratives” around the Sputnik V vaccine and several Russia-developed COVID-19 treatments.

As shown in Fig 5, our analysis yields an interesting finding about Russian unverified sources’ coverage of vaccine-related content. Russian unverified sources were among the main spreaders of “anti-vax narratives”—in our dataset, Russia ranked as the third source country for the spread of “anti-vax narratives,” after the US and Germany. At the same time, Russian unverified sources had the highest number of articles promoting “pro-Russia narratives” around the Sputnik V vaccine and several Russia-developed COVID-19 treatments.

**Fig 6:**
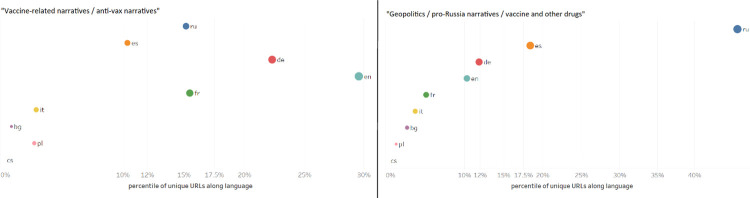
Focus on Russian unverified sources. The scatter plot shows the language coverage of all articles by Russian unverified sources annotated as “vaccine-related narratives / anti-vax narratives” (01 January 2020–31 December 2022) and all articles by Russian unverified sources annotated as “geopolitics / pro-Russia narratives / vaccine and other drugs” (01 January 2020–31 December 2022).

As shown in Fig 6, Russian unverified sources spread anti-vax narratives mainly in foreign languages. English, German, and French articles comprised around 67 per cent of all articles from Russian unverified sources annotated as spreading “anti-vax narratives.” In comparison, only 15 per cent of articles from Russian unverified sources annotated as spreading “anti-vax narratives” were in Russian. This indicates that Russian unverified sources were mainly focused on spreading anti-vax narratives among foreign audiences.

On the other hand, Russian unverified sources promoted the Sputnik V vaccine both in Russian and in a variety of foreign languages. 45 per cent of the articles annotated as “geopolitics / pro-Russia narratives / vaccines and other drugs” were in Russian. The remaining 55 per cent was, in descending order, in Spanish, German, English, French, Italian, Bulgarian, Polish, and Czech. This indicates that Russian unverified sources were focused on promoting the Sputnik V vaccine both at home and abroad. An in-depth analysis of the data, moreover, shows that Russian unverified sources portrayed the Sputnik V vaccine as an instrument for the Kremlin to demonstrate international solidarity and assert itself as the global leader that would save the world from the pandemic. This is in line with existing research finding that the Kremlin used the Sputnik V vaccine as a tool to strengthen its influence abroad [51].

**Fig 7:**
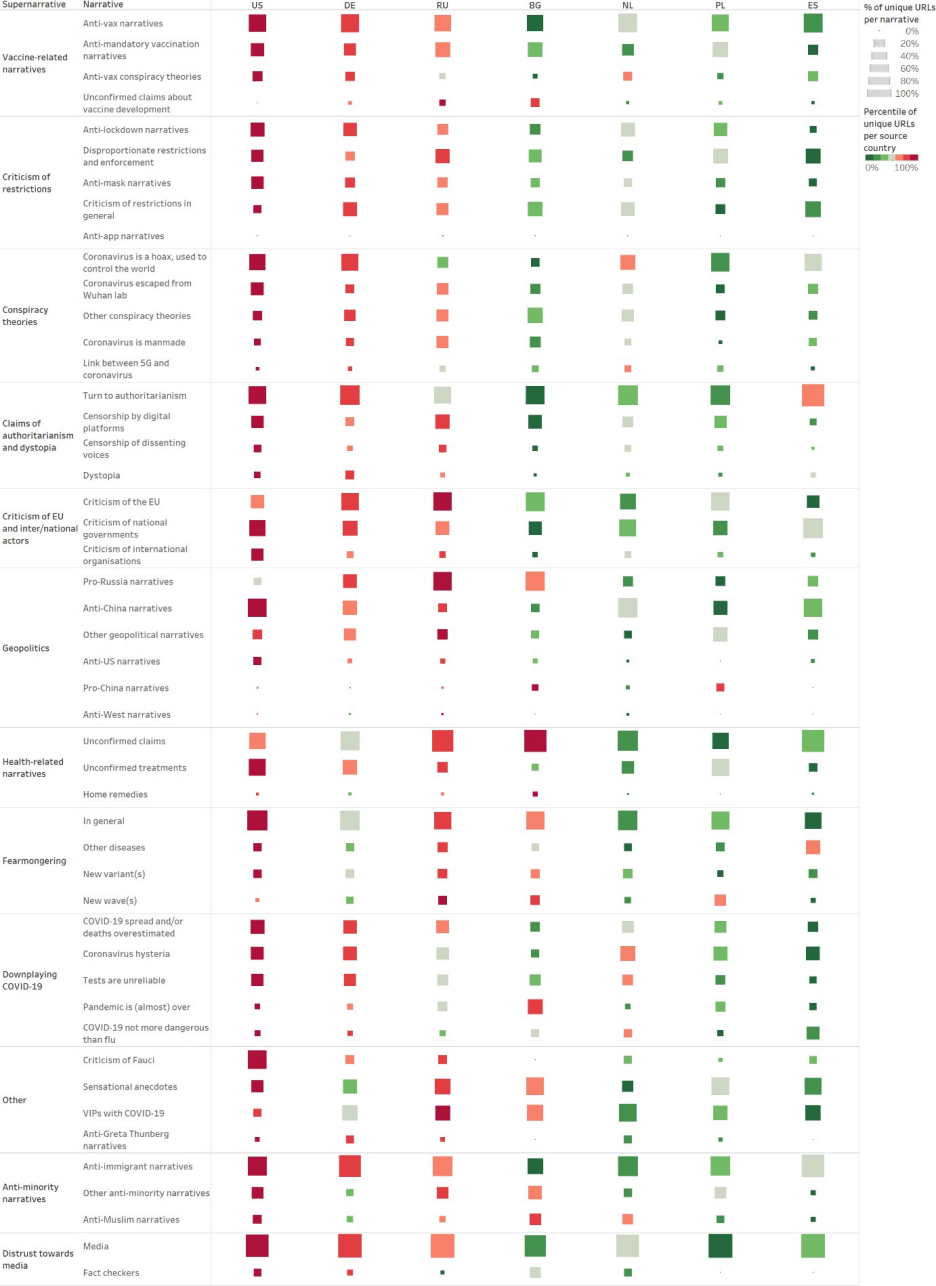
Supernarrative country distribution. The table shows the country distribution of all supernarratives and narratives. The bigger the square, the more articles from the monitored sources of a given country have been assigned to the narrative in question. The colour represents the distribution of articles in percentile across the narrative. Using percentile allows us to display each source country’s ranking within each narrative, despite the different numbers of monitored sources for each country.

Having analysed the main mis/disinformation narratives around COVID-19 and their spread over time, we now proceed to analyse country-specific trends, as shown in Fig 7. As we noted above, one of the main limitations of our study is the uneven country coverage of our list of unverified sources, which contains many American, Russian, and Western European sources compared to a lower number of sources from many Eastern European countries. Taking this limitation into account, we normalised the data, in order to make the results comparable. Our country-based analysis focuses on the top seven countries for which we annotated a significant number of articles. These are the United States, Germany, Russia, Bulgaria, the Netherlands, Poland, and Spain. The annotated data from these seven countries represent over 80 per cent of the whole dataset.

Looking at the distribution across the top seven countries for all supernarratives and narratives, we identified several outliers.

Within the “criticism of restrictions” supernarrative, German and Russian unverified sources argued against all types of restrictions almost equally, while Spanish unverified sources predominantly focused on spreading claims that restrictions and their enforcement were disproportionate to the danger posed by COVID-19.

Concerning “conspiracy theories,” German, Dutch, and Polish unverified sources mainly spread allegations that the “coronavirus is a hoax, used to control the world,” while Russian unverified sources mainly spread the conspiracy theory that the novel “coronavirus escaped from Wuhan lab” or general theories that the “coronavirus is manmade.”

Within the “claims of authoritarianism and dystopia” supernarrative, Spanish unverified sources were especially vocal about the “authoritarian” aspect of the pandemic, arguing that there is a “connection between contagious diseases and authoritarianism.” They claimed that the “coronavirus brought a neo-communist regime to Spain” and accused the “social-communist government” of implementing policies that led to “a de facto dictatorship.”

Within the “criticism of [the] EU and inter/national actors” supernarrative, US unverified sources strongly pushed “criticism of national” but also “criticism of international organisations,” mainly of the WHO, supporting the Trump administration’s decision to halt WHO funding in 2020 and accusing the UN agency of being under China’s control. Russian and European unverified sources, on the other hand, were the main spreaders of “criticism of the EU.” Russian and German unverified sources published, respectively, 20 and 15 per cent of all articles annotated as “criticism of the EU,” followed by Polish, Bulgarian, Dutch and Spanish unverified sources.

Within the “geopolitics” supernarrative, Russian unverified sources were the main spreaders of “pro-Russia narratives,” followed by Bulgarian unverified sources. On the other hand, US unverified sources were the main spreaders of “anti-China narratives,” followed by German and Dutch unverified sources. “Pro-China narratives” represented only 0.1 per cent of the whole dataset. However, it is interesting to note that they were mainly spread by Bulgarian and Polish unverified sources. Bulgarian unverified sources praised China for sending medical equipment to Bulgaria and for having successfully developed a COVID-19 vaccine and supplied it to neighbouring Serbia. Polish unverified sources widely reported that the Polish government was interested in buying the Chinese vaccine and described the cooperation with China as a strategic partnership.

Within the “health-related” supernarrative, Bulgarian unverified sources published half of all articles annotated as related to “home remedies.” These articles claimed that the consumption of yoghurt, whiskey, and different types of herbal teas would prevent and/or cure COVID-19.

**Fig 8:**
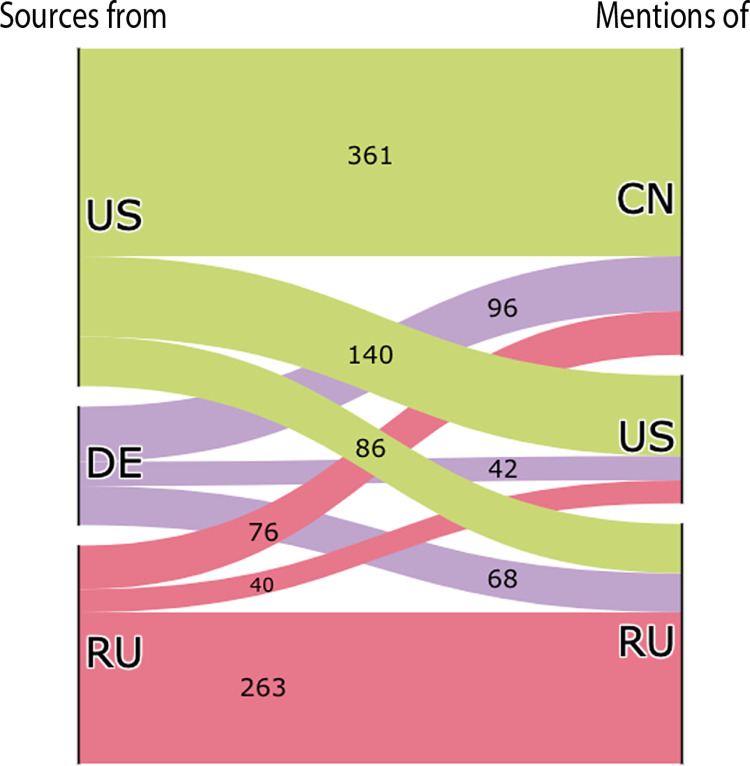
Sankey diagram. The Sankey Diagram shows the link between the source countries of unverified sources (US, Germany, and Russia were selected) and the countries mentioned in the articles annotated as related to “geopolitics”.

The Sankey diagram below (Fig 8) offers a different perspective on country-based reporting concerning the “geopolitics” supernarrative. Amongst the articles that have been annotated as related to geopolitics, it is possible to identify the geopolitical focus of the unverified sources of a given country by analysing the link between the source country of unverified sources and the countries mentioned in the articles via our internal GeoRSS toponym detection system. Given our bias in the country coverage, we focus our analysis on the three source countries (US, Russia, Germany–shown on the left in Fig 8) for which we annotated the highest numbers of articles as related to “geopolitics.”

As Fig 8 clearly shows, US unverified sources mainly focused on China. Our data indicate that this is a negative focus, as US unverified sources spread several anti-China narratives, accusing China of being responsible for the pandemic, of being intransparent and untrustworthy in its public health communications, and of delivering faulty medical equipment to countries in need. Echoing former US President Donald Trump’s words, US unverified sources often referred to the SARS-CoV-2 virus as the “Chinese virus” or “Kung-Flu.” The sustained use of this language well passed the time in which the virus was mainly concentrated in China suggests that the expression was motivated by its desired geopolitical implications rather than geography. US unverified sources focused much less on the US and even less on Russia. Our data indicate that when US unverified sources turned their focus inward, they mainly accused the US administration of mismanaging the health crisis, both at federal and state level.

German unverified sources also mainly focused on China, accusing Xi Jinping’s government of being responsible for the spread of the coronavirus due to its intransparent handling of the initial health crisis. Our data show a concentration of such articles in April/May 2020. Further analysis suggests that the peak was triggered by an article by German tabloid newspaper *Bild* [52], which argued that China owed Germany 149 billion EUR due to lost revenue during the first couple of months of the pandemic; unverified sources falsely reported that Germany had sent China a bill of 149 billion euros for “coronavirus damages.”

Finally, Russian unverified sources mainly focused on Russia itself, spreading a wide range of pro-Russia narratives: they boasted about the development and deployment of the Sputnik V vaccine and several COVID-19 treatments, they praised Russia’s medical solidarity to countries in need, and they accused the West of being russophobic and uncollaborative, especially in its rejection of the Sputnik V vaccine. To a lesser extent, Russian unverified sources also focused on China. Our data show that Russian unverified sources’ approach to China was ambivalent: while most of the articles spread similar anti-China narratives to those spread by US and German unverified sources—e.g. that China was responsible for the pandemic, it could not be trusted, etc.—some articles also spread some pro-China narratives, praising Chinese efforts in COVID-19 vaccine development and deployment, and in international aid to countries in need.

It is interesting to note that a significant amount of geopolitically themed articles by US, German, and Russian unverified sources focused on China. While the initial focus on China can be justified by the fact that the first COVID-19 outbreak was detected in Wuhan, the sustained focus on China and an in-depth analysis of the main narratives suggests that unverified sources leveraged the pandemic to spread anti-China sentiment. Unsurprisingly, Russian unverified sources also leveraged the pandemic to spread pro-Russia sentiment.

**Fig 9:**
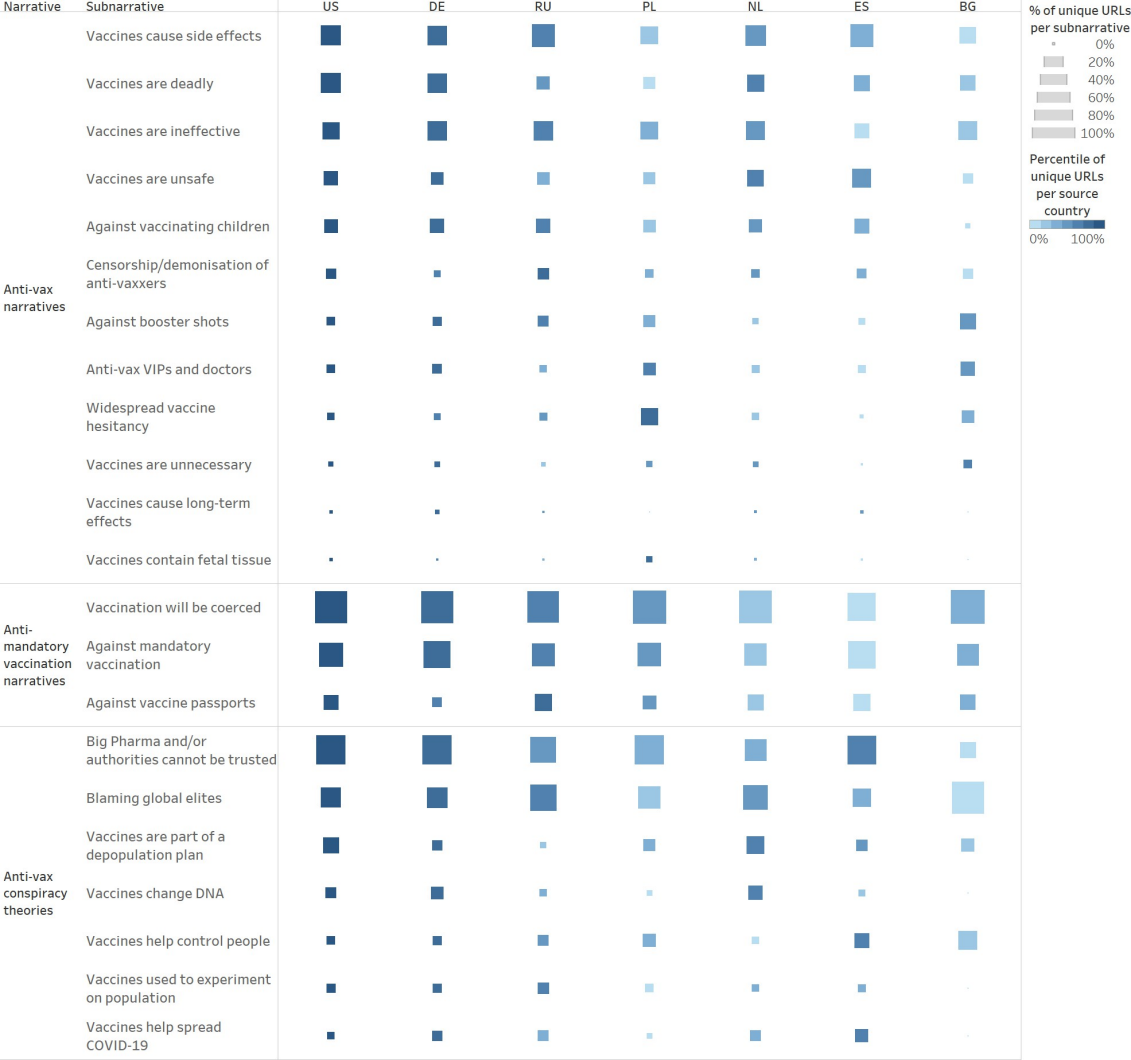
Country distribution for anti-vax narratives. The table shows the country distribution of all anti-vax narratives and subnarratives. The colour of the square indicates the country’s ranking based on the total number of articles per subnarrative—the darkest square represents the country with the highest percentile of articles per subnarrative, etc. The size of the square indicates the normalised number of articles per country per subnarrative, that is, the number of articles tagged as each subnarrative compared to the total number of monitored sources per country—the bigger the square, the more articles from the monitored sources of a given country have been assigned to the subnarrative in question.

Having analysed country-specific trends regarding supernarratives and narratives and having delved deeper into country-based reporting concerning the “geopolitics” supernarrative, we now proceed to analyse country-specific trends regarding anti-vax narratives and subnarratives, as shown in Fig 9. Similarly to our country-based analysis of supernarratives and narratives, we normalised the data in order to make the results comparable.

Given the constraints of our database of unverified sources, it is not surprising that in Fig 9, the US is consistently associated with the darkest square—the US is the country with the highest percentile of articles per anti-vax subnarrative because it is the country for which we monitored the highest number of unverified sources. Similarly, Germany and Russia often present the second and third darkest squares because they were the second and third countries for numbers of monitored sources.

It is far more interesting to compare the sizes of the squares in Fig 9, as the size of the square indicates the normalised number of articles per country per subnarrative, given the numbers of monitored sources per country. While most of the anti-vax subnarratives appear to be similarly widespread across the analysed countries, there are four subnarratives that appear to be more widespread in some countries than others.

The “against booster shots” subnarrative was particularly widespread among Bulgarian sources. According to data from the European Centre for Disease Prevention and Control (ECDC), Bulgaria has the lowest COVID-19 vaccination rate in the EU with only 30 per cent of its population having completed the primary course of vaccination and only 12 per cent having received a booster shot [53]. It is plausible that the anti-vax claims spread by Bulgarian unverified sources had a direct effect on the Bulgarian population’s willingness to get vaccinated and boosted.

The “anti-vax VIPs and doctors” subnarrative was particularly popular among Bulgarian and Polish unverified sources. This is not surprising given that in both Bulgaria and Poland, many politicians, celebrities and doctors expressed vaccine scepticism. There are plenty of news reports on Bulgarian doctors who “aren’t sure COVID-19 vaccines are a good idea” [54], Polish politicians “flirting” with anti-vax voters [55] and Polish celebrities “expressing reservations” about COVID-19 vaccines [56], etc.

The “widespread vaccine hesitancy” subnarrative was especially widespread among Polish unverified sources. Throughout 2020, Polish unverified sources reported on several surveys that were carried out before COVID-19 vaccines became available and found that a large segment of the Polish population was not willing to get vaccinated against COVID-19.

Finally, conspiracy theories “blaming global elites” and alleging that “vaccines help control people” were especially popular among Bulgarian unverified sources. Throughout 2020 and 2021, Bulgarian unverified sources published several articles accusing Bill Gates of being “behind everything.”

## Discussion

The analysis of the annotated data shows how mis/disinformation narratives emerged and developed during the pandemic, often following factual events. Of particular interest is the rise of anti-vax content in November 2020, as preliminary data on the effectiveness of COVID-19 vaccines became available. Vaccine-related mis/disinformation may open the door to pseudoscience, such as homeopathy and other so-called “alternative medicine” methods, and outright dangerous charlatanry, such as drinking bleach. Our findings about anti-vax narratives and false cures align with what the literature evidenced about the spread of mis/disinformation within European countries. Indeed, Siwakoti and colleagues showed that narratives supporting anti-vax claims and promoting false remedies to prevent infection and cure the symptoms of COVID-19 were of particular relevance in Europe [15]. Similarly to what Darius and Urquhart concluded about COVID-19 mis/disinformation leading to the deterioration of public trust on Twitter [14], the results of our analysis suggest that irrational behaviour contradicting official statements—such as the refusal to get vaccinated in spite of the scientific evidence showing the benefits of vaccination and the willingness of some individuals to “self-medicate” with products that have not been scientifically proven to be effective—can undermine trust in medicine as a scientific discipline as well as in public health authorities and their expertise within democratic societies. Thus, we recommend further research into mis/disinformation narratives, their evolution, and outreach, in view of developing efficient public health communication as similarly indicated by Clemente-Suárez and colleagues [11]. The argument in favour of improved public health communication also considers what previous research showed about the persistence of concepts and ideas conveyed by disinformation content in individuals’ minds [9].

In comparison to previous academic research addressing the issue of mis/disinformation narratives related to COVID-19 [11, 14], our methodological approach based on the multi-level codebook allowed for the detailed annotation of the high number of text items encountered during the infodemic and built the basis for training a Transformer-model to reduce the burden of manual annotations. Even though previous studies similarly adopted codebooks to frame and investigate COVID-19 disinformation narratives [10, 15], the classification by the Transformer-model that we adopted in our research led to a significant reduction of time required for daily work, allowing us to analyse a larger size of data and building the base for further quantitative and qualitative analysis.

Furthermore, filtering by language or source country showed differences pointing to country-specific trends. This was particularly useful for analysing events of geopolitical interest, such as the promotion of the Sputnik V vaccine termed as vaccine diplomacy. These findings align with what Siwakoti and colleagues evidenced in their research identifying country-related disinformation narratives [15] as well as the geopolitical framing of vaccines by state actors. Acknowledging the benefit of this methodology, we recommend this approach in further research aimed at identifying different portrayals of events among unverified sources from several countries and at spotting disinformation for political gain.

## Conclusions

The detailed COVID-19 codebook enabled the study of long-term mis/disinformation trends during the first three years of the coronavirus pandemic. Manual annotations were used to build a training set for a transformer-based model. The release of the codebook in the supporting information, a product of three years of work, will empower experts working on mis/disinformation to continue the trend analysis as, unfortunately, we are still facing the pandemic and its accompanying infodemic. The present work will also be useful for the development of additional codebooks to study vaccine hesitancy, future epidemics, and other public health events.

Furthermore, the transformer-based model can be used as blueprint to study other topics around which a large volume of mis/disinformation can be found. Work is ongoing to analyse mis/disinformation on climate change, migration and geopolitical issues, based on codebooks, training sets and transformer-based models.

While an experienced analyst can identify trends as part of daily duties, our approach quantifies trends over time, e.g. the rise of anti-vax content in parallel to the development and approval of vaccines against SARS-CoV-2. It also allows for the comparison of the spread of mis/disinformation narratives with general news content, e. g. from Europe Media Monitor (EMM). Indeed, the COVID-19 pandemic has demonstrated that many mis/disinformation narratives are based on factual events that are misrepresented or taken out of context.

## Supporting information

S1 File Query. Keyword-based query. (PDF)

S2 File COVID-19 codebook. The COVID-19 codebook has three levels: 12 supernarratives, 51 narratives, and 44 subnarratives. Each supernarrative features several narratives; narratives may further contain subnarratives. The supernarratives are organised based on article count in descending order, so are the narratives within a supernarrative and the subnarratives within a narrative. Each code presents a general description and a specific example drawn from our dataset.
(PDF)

S1 Table Detailed results of the classifier per supernarrative. The table shows precision, recall and F1 score for the supernarratives.
(PDF)

S1 Fig Country source coverage. The map illustrates the country coverage of our database of 460 unverified sources, clearly showing that the databased of unverified sources this investigation is based on contains many US, Western European, and Russian sources while it is lacking Latin American, African, and Asian sources.
(TIF)

S2 Fig Evolution of anti-vax narratives. The timeline shows the spread over time of “anti-vax narratives,” “anti-mandatory vaccination narratives,” and “anti-vax conspiracy theories”.
(TIF)

S3 Fig Overall timeline. The timeline shows the overall distribution of all annotated COVID-19 related data.
(TIF)

## References

[1] World Health Organization. Epidemic intelligence from open sources (EIOS). Sav Lives Early Detect Available Online Httpswww Who Inteios Accessed May 14 2020. 2020;

[2] Do Nascimento IJB, Pizarro AB, Almeida JM, Azzopardi-Muscat N, Gonçalves MA, Björklund M, et al. Infodemics and health misinformation: a systematic review of reviews. Bull World Health Organ. 2022;100(9):544. doi: 10.2471/BLT.21.287654 36062247 PMC9421549

[3] Apetrei C, Marx PA, Mellors JW, Pandrea I. The COVID misinfodemic: not new, never more lethal. Trends Microbiol. 2022; 10.1016/j.tim.2022.07.004 PMC9356696 35945120

[4] Brennen JS, Simon FM, Howard PN, Nielsen RK. Types, sources, and claims of COVID-19 misinformation [PhD Thesis]. University of Oxford; 2020.

[5] Gisondi MA, Barber R, Faust JS, Raja A, Strehlow MC, Westafer LM, et al. A deadly infodemic: Social media and the power of COVID-19 misinformation. Vol. 24, Journal of medical Internet research. JMIR Publications Toronto, Canada; 2022. p. e35552. doi: 10.2196/35552 PMC8812140 35007204

[6] European Commission. Code of Practice on Disinformation. September; 2018.

[7] European Commission. Guidance to strengthen Code of Practice on Disinformation. 2021.

[8] European Commission. The Strengthened Code of Practice on Disinformation 2022. 2022.

[9] Ecker UK, Lewandowsky S, Cook J, Schmid P, Fazio LK, Brashier N, et al. The psychological drivers of misinformation belief and its resistance to correction. Nat Rev Psychol. 2022;1(1):13–29.

[10] León B, Martínez-Costa MP, Salaverría R, López-Goñi I. Health and science-related disinformation on COVID-19: A content analysis of hoaxes identified by fact-checkers in Spain. PloS One. 2022;17(4):e0265995. doi: 10.1371/journal.pone.0265995 35417493 PMC9007356

[11] Clemente-Suárez VJ, Navarro-Jiménez E, Simón-Sanjurjo JA, Beltran-Velasco AI, Laborde-Cárdenas CC, Benitez-Agudelo JC, et al. Mis–dis information in covid-19 health crisis: a narrative review. Int J Environ Res Public Health. 2022;19(9):5321. doi: 10.3390/ijerph19095321 35564714 PMC9101334

[12] Agley J, Xiao Y. Misinformation about COVID-19: evidence for differential latent profiles and a strong association with trust in science. BMC Public Health. 2021;21:1–12. 33413219 10.1186/s12889-020-10103-x PMC7789893

[13] Evanega S, Lynas M, Adams J, Smolenyak K, Insights CG. Coronavirus misinformation: quantifying sources and themes in the COVID-19 ‘infodemic’. JMIR Prepr. 2020;19(10):2020.

[14] Darius P, Urquhart M. Disinformed social movements: A large-scale mapping of conspiracy narratives as online harms during the COVID-19 pandemic. Online Soc Netw Media. 2021;26:100174. doi: 10.1016/j.osnem.2021.100174 34642647 PMC8495371

[15] Siwakoti S, Yadav K, Thange I, Bariletto N, Zanotti L, Ghoneim A, et al. Localized misinformation in a global pandemic: Report on COVID-19 narratives around the world. Empir Stud Confl. 2021;

[16] Adebayo AL, Davidson Mhonde R, DeNicola N, Maibach E. The effectiveness of narrative versus didactic information formats on pregnant women’s knowledge, risk perception, self-efficacy, and information seeking related to climate change health risks. Int J Environ Res Public Health. 2020;17(19):6969. doi: 10.3390/ijerph17196969 32977683 PMC7579394

[17] Carrion ML. “You need to do your research”: Vaccines, contestable science, and maternal epistemology. Public Underst Sci. 2018;27(3):310–24. doi: 10.1177/0963662517728024 28841813

[18] Coan TG, Boussalis C, Cook J, Nanko MO. Computer-assisted classification of contrarian claims about climate change. Sci Rep. 2021;11(1):22320. doi: 10.1038/s41598-021-01714-4 34785707 PMC8595491

[19] Shorten C, Khoshgoftaar TM, Furht B. Deep Learning applications for COVID-19. J Big Data. 2021;8(1):1–54. 33457181 10.1186/s40537-020-00392-9 PMC7797891

[20] Meddeb P, Ruseti S, Dascalu M, Terian SM, Travadel S. Counteracting french fake news on climate change using language models. Sustainability. 2022;14(18):11724.

[21] Hossain T, Logan IV RL, Ugarte A, Matsubara Y, Young S, Singh S. COVIDLies: Detecting COVID-19 misinformation on social media. In: Workshop on NLP for COVID-19 (Part 2) at EMNLP 2020. 2020.

[22] Schmidt A, Ivanova A, Schäfer MS. Media attention for climate change around the world: A comparative analysis of newspaper coverage in 27 countries. Glob Environ Change. 2013;23(5):1233–48.

[23] Kouzy R, Abi Jaoude J, Kraitem A, El Alam MB, Karam B, Adib E, et al. Coronavirus goes viral: quantifying the COVID-19 misinformation epidemic on Twitter. Cureus. 2020;12(3). doi: 10.7759/cureus.7255 32292669 PMC7152572

[24] Wetts R Models and morals: Elite-oriented and value-neutral discourse dominates American organizations’ framings of climate change. Soc Forces. 2020;98(3):1339–69.

[25] Zhang C, Gupta A, Kauten C, Deokar AV, Qin X. Detecting fake news for reducing misinformation risks using analytics approaches. Eur J Oper Res. 2019;279(3):1036–52.

[26] Dictionary C. Narrative [Internet]. Available from: https://dictionary.cambridge.org/it/dizionario/inglese/narrative

[27] Devlin J, Chang MW, Lee K, Toutanova K. Bert: Pre-training of deep bidirectional transformers for language understanding. ArXiv Prepr ArXiv181004805. 2018;

[28] Johns Hopkins Center for Systems Science and Engineering. Coronavirus COVID-19 Global Cases. 2020.

[29] Lufkin B. Coronavirus: The psychology of panic buying [Internet]. 2020. Available from: https://www.bbc.com/worklife/article/20200304-coronavirus-covid-19-update-why-people-are-stockpiling

[30] Faulconbridge G. George Soros says EU may not survive coronavirus crisis [Internet]. 2020. Available from: https://www.reuters.com/article/us-health-coronavirus-eu-soros/george-soros-says-coronavirus-threatens-eus-survival-idUSKBN22Y168

[31] EMA recommends approval of Comirnaty and Spikevax COVID-19 vaccines for children from 6 months of age [Internet]. 2022. Available from: https://www.ema.europa.eu/en/news/ema-recommends-approval-comirnaty-spikevax-covid-19-vaccines-children-6-months-age

[32] Ongoing EPPO investigation into the acquisition of COVID-19 vaccines in the EU [Internet]. 2022. Available from: https://www.eppo.europa.eu/en/news/ongoing-eppo-investigation-acquisition-covid-19-vaccines-eu

[33] Protests against COVID measures across Europe [Internet]. 2021. Available from: https://www.reuters.com/news/picture/protests-against-covid-measures-across-e-idUSRTS3WWZG

[34] WHO chief calls for ‘audits’ of Wuhan labs after first mission controversy [Internet]. 2021. Available from: https://www.theguardian.com/world/2021/jul/16/who-chief-calls-for-audits-of-wuhan-labs-after-first-mission-controversy

[35] Pfizer B. Pfizer and BioNTech announce vaccine candidate against COVID-19 achieved success in first interim analysis from phase 3 study. Pfizer N Y NY USA. 2020;

[36] Covid-19 vaccine: First person receives Pfizer jab in UK [Internet]. 2020. Available from: https://www.bbc.com/news/uk-55227325

[37] U.S. Starts Vaccine Rollout as High-Risk Health Care Workers Go First [Internet]. 2021. Available from: https://www.nytimes.com/live/2020/12/14/world/covid-19-coronavirus

[38] European Commission. European Commission authorises first safe and effective vaccine against COVID-19 [Internet]. 2020. Available from: https://ec.europa.eu/commission/presscorner/detail/en/ip_20_2466

[39] Food US and Administration Drug. Coronavirus (COVID-19) update: FDA authorizes Pfizer-BioNTech COVID-19 vaccine for emergency use in adolescents in another important action in fight against pandemic. US Food Drug Adm Silver Spring MD USA. 2021;

[40] US Food and Drug Administration. Coronavirus (COVID-19) Update: FDA Authorizes Additional Vaccine Dose for Certain Immunocompromised Individuals [Internet]. 2021. Available from: https://www.fda.gov/news-events/press-announcements/coronavirus-covid-19-update-fda-authorizes-additional-vaccine-dose-certain-immunocompromised

[41] Reviews of articles by: Peter McCullough. Available from: https://healthfeedback.org/authors/peter-mccullough/

[42] Alba. The Latest Covid Misinformation Star Says He Invented the Vaccines. N Y Times [Internet]. 2022; Available from: https://www.nytimes.com/2022/04/03/technology/robert-malone-covid.html

[43] Musk E. Based on current trends, probably close to zero new cases in US too by end of April [Internet]. 2020. Available from: https://twitter.com/elonmusk/status/1240754657263144960

[44] Musk E. Kids are essentially immune, but elderly with existing conditions are vulnerable. Family gatherings with close contact between kids & grandparents probably most risky. [Internet]. 2020. Available from: https://twitter.com/elonmusk/status/1240758710646878208

[45] Musk E. Why aren’t they dead yet? [Internet]. 2021. Available from: https://twitter.com/elonmusk/status/1476437555717541893

[46] Levitan. Paul Repeats Baseless Vaccine Claims. FactCheck [Internet]. 2015; Available from: https://www.factcheck.org/2015/02/paul-repeats-baseless-vaccine-claims/

[47] Kaczynski A. Ron Johnson said last fall that undermining the Covid vaccine ‘will cause people’s deaths.’ Now he spreads anti-vaccine misinformation. CNN [Internet]. 2021; Available from: https://edition.cnn.com/2021/09/09/politics/ron-johnson-vaccine-kfile/index.html

[48] Kosc W. Polish president taps into anti-vax sentiment ahead of elections [Internet]. 2020. Available from: https://www.politico.com/news/2020/07/09/poland-andrzej-duda-anti-vaccination-coronavirus-355016

[49] European Commission. Questions and Answers–Digital Green Certificate [Internet]. 2021. Available from: https://ec.europa.eu/commission/presscorner/detail/en/qanda_21_1187

[50] COVID-19 Portal [Internet]. Available from: https://www.salute.gov.it/portale/nuovocoronavirus/homeNuovoCoronavirus.jsp?lingua=italiano

[51] Michlin-Shapir V, Khvostunova O. The rise and fall of Sputnik V: How the Kremlin used the coronavirus vaccine as a tool of information warfare. Inst Mod Russ [Internet]. 2021; Available from: https://imrussia.org/en/news/3358-the-rise-and-fall-of-sputnik-v%E2%80%94a-new-report-by-imr

[52] Was China uns jetzt schon schuldet [Internet]. 2020. Available from: https://www.bild.de/bild-plus/politik/ausland/politik-ausland/bild-praesentiert-die-corona-rechnung-was-china-uns-jetzt-schon-schuldet-70044300.bild.html

[53] Prevention EC for D, Control. COVID-19 Vaccine Tracker. Available from: https://vaccinetracker.ecdc.europa.eu/public/extensions/COVID-19/vaccine-tracker.html#uptake-tab

[54] Ivanova M. In Bulgaria, even doctors aren’t sure COVID-19 vaccines are a good idea [Internet]. 2021. Available from: https://www.euronews.com/my-europe/2021/05/18/in-bulgaria-even-doctors-aren-t-sure-covid-19-vaccines-are-a-good-idea

[55] Wanat Z. Poland’s vaccine skeptics create a political headache [Internet]. 2021. Available from: https://www.politico.eu/article/poland-vaccine-skeptic-vax-hesitancy-political-trouble-polish-coronavirus-covid-19/

[56] Sieradzka M. Anti-vaccine sentiment rife in Poland [Internet]. 2020. Available from: https://www.dw.com/en/anti-vaccine-sentiment-rife-in-poland/a-56100878

