# Supplementary material for: Trend analysis of COVID-19 mis/disinformation narratives–A 3-year study
Source: PLoS One. 2023 Nov 17;18(11):e0291423. doi: 10.1371/journal.pone.0291423 (PMC10655972; doi:10.1371/journal.pone.0291423)
Supplement: S2 File — The COVID-19 codebook has three levels: 12 supernarratives, 51 narratives, and 44 subnarratives. Each supernarrative features several narratives; narratives may further contain subnarratives. The supernarratives are organised based on article count in descending order, so are the narratives within a supernarrative and the subnarratives within a narrative. Each code presents a general description and a specific example drawn from our dataset. (PDF) [file pone.0291423.s002.pdf]

**Supernarrative**	**Narrative**	**Subnarrative**	**Description**
Vaccine-related narratives	Anti-vax narratives	Vaccines cause side effects	Vaccines are likely to cause serious side effects, ranging from scientifically recognised ones, such as blood clots, to ones that do not have any scientific basis, such as infertility and cancer.
			E.g. “Slovakia has detected a large number of suspected side effects after the COVID-19 vaccine!”
		Vaccines are deadly	Vaccines might kill you. Many vaccinated individuals have died after vaccination.
			E.g. “Horror! Two more people died after receiving the Pfizer/BioNTech vaccine”
		Vaccines are ineffective	Vaccines are ineffective. They will not work against new strains of coronaviruses; vaccinated individuals have contracted and died of COVID-19, *etc*.
			E.g. “Robert Koch Institute report released today states that 95.58 per cent of the #Omicron cases in Germany are fully vaccinated”
		Vaccines are unsafe	Vaccines are unsafe because they were not tested properly, they contain dangerous ingredients, such as graphene, *etc*.
			E.g. “Victim of experimental COVID-19 vaccine says he has ‘never been so sick in his life' after being injected with experimental vaccine”
		Against vaccinating children	Children should not be vaccinated. Vaccines are dangerous and they are not worth the risk, especially for children, who are barely affected by COVID-19.
			E.g. “Vaccinating children against COVID is irresponsible and unethical”
		Censorship / demonisation of anti-vaxxers	Claims that individuals who refuse to get vaccinated are censored through authoritarian means or demonised

			E.g. “California man thrown in psychiatric hold for expressing concern about Bill Gates’ vaccines”
		Against booster shots	Booster shots, which are also dangerous, will be required repeatedly.
			E.g. “People will have to ‘get used’ to endless booster vaccinations”
		Anti-vax VIPs and doctors	Claims by politicians, influencers and doctors that are either anti-vax or misconstrued as such
			E.g. ““SpaceX and Tesla founder Elon Musk has said that neither he nor his family will likely take future coronavirus vaccines”
		Widespread vaccine hesitancy	Biased reporting on widespread vaccine hesitancy
			E.g. “1 in 2 Italians does not want to get vaccinated […] Operation terror does not seem to have worked”
		Vaccines are unnecessary	Vaccines are unnecessary or even pointless. A herd immunity approach and/or a treatment would be preferable.
			E.g. “‘All infected people develop antibodies’ […] The vaccine is, therefore, not needed. Herd immunity can be achieved by infecting the population in a controlled way”
		Vaccines cause long-term effects	The long-term effects of COVID-19 vaccines are unknown. Thus, we cannot exclude that COVID-19 vaccines will have negative long-term effects.
			E.g. “COVID-19 shots could reduce life expectancy by several decades”
		Vaccines contain fetal tissue	COVID-19 vaccines contain fetal tissue and that is unethical.
			E.g. “‘Scandalous’ Vatican: ‘Yes to vaccines obtained from aborted fetuses”
			Any other anti-vax narratives

		Other anti-vax narratives	E.g. “Vaccines against COVID-19 kill sharks: what is squalene and what alternatives are there?”
	Anti-mandatory vaccination narratives	Vaccination will be coerced	Vaccination will be coerced one way or another - if not by making it mandatory, by making life impossible for those who refuse to get vaccinated until they are forced to change their minds.
			E.g. “German Geneticist Says People Who Refuse COVID Vaccine Should Be Denied Medical Treatment”
		Against mandatory vaccination	Mandatory vaccination infringes upon fundamental rights.
			E.g. “There is no legal, moral or logical argument that justifies government entities demanding access to your body in order to inject foreign substances into you”
		Against vaccine passports	Vaccine passports should be banned as they infringe upon individual rights and discriminate against unvaccinated individuals.
			E.g. “VACCINE PASSPORTS are 100 per cent about COERCION and NOT disease, medicine or science”
	Anti-vax conspiracy theories	Big Pharma and / or authorities cannot be trusted	Big Pharma and/or world governments cannot be trusted with the development and distribution of vaccines as they have their own dark agendas.
			E.g. “BOMBSHELL Cover-up: Dr Anthony Fauci helped approve an effective treatment for coronavirus infections 15 years ago, but is suppressing it today in favour of new high-profit vaccines”
		Blaming global elites	Vaccines are the product of a dark scheme by the global elites (Bill Gates, George Soros, the Rockefellers, *etc*.).
			E.g. “Coronavirus reveals links between China, Sanofi, George Soros and Bill Gates”

		Vaccines are part of a depopulation plan	Vaccines are aimed at reducing the world population.
			E.g. “DEPOPULATION VAX: Trial subjects injected with coronavirus vaccines suddenly test positive for HIV”
		Vaccines change DNA	mRNA vaccines alter people’s DNA.
			E.g. “New COVID-19 Vaccine Is Untested and Will Alter Your DNA”
		Vaccines help control people	Vaccines are an instrument through which the elites plan to control the masses (e.g. vaccines contain microchips).
			E.g. “Trump's former adviser suggests that Bill Gates would have created the Coronavirus to implant microchips in the world's population”
		Vaccines used to experiment on population	Vaccines are first tested on Africans or African Americans or are part of a global experiment carried out by the elites.
			E.g. “Gates We Are Not Your Lab Rats”: Africans Protest COVID-19 Vaccine Testing”
		Vaccines help spread COVID-19	COVID-19 vaccines facilitate coronavirus infections.
			E.g. “They tell us about the fifth wave but what they don't tell us [...] is that the fifth wave is caused by the vaccines”
		Other anti-vax conspiracy theories	Any other anti-vax conspiracy theories
			E.g. “Coronavirus vaccine delayed until researcher can figure out how to get it to cause autism”
	Unconfirmed claims about vaccine development		Unreliable claims that a (new) COVID-19 vaccine is being developed
			E.g. “A Texas-based company reportedly developed a coronavirus vaccine” (February 2020)
Criticism of restrictions	Anti-lockdown narratives		Lockdowns are ineffective at reducing the spread of COVID-19; lockdowns

			have bigger negative effects on people's health than COVID-19 does.
			E.g. "Coronavirus lockdown: We are so afraid of death, no one even asks whether this 'cure' is actually worse"
	Disproportionate restrictions and enforcement		Coronavirus restrictions and their enforcement by the authorities are exaggerated, disproportionate to the actual threat posed by the epidemic.
			E.g. "British Police Shut Down Good Friday Service, Threatening Worshippers with Fines and Arrest"
	Anti-mask narratives		Masks are ineffective at reducing the spread of COVID-19; masks have bigger negative effects on people's health than COVID-19 does.
			E.g. "Sweden's Top Doctor says Face Masks are Very Dangerous"
	Criticism of restrictions in general		Criticism of restrictions in general or of any other restrictions, such as quarantine, social distancing, *etc*.
			E.g. "The 1.5 metre society must end as soon as possible"
	Anti-app narratives		Contact-tracing apps are ineffective at reducing the spread of COVID-19.
			E.g. "Infectious disease specialist: corona app is relatively useless"
Conspiracy theories	Coronavirus is a hoax, used to control the world		The coronavirus pandemic is a hoax. It is part of "The Great Reset" - a plan concocted by the elites to exert further control over the masses.
			E.g. "Elites Are Pushing COVID Lockdowns to Usher in 'The Great Reset'"
	Coronavirus escaped from Wuhan lab		The coronavirus was created at and escaped from the Wuhan Institute of Virology.
			E.g. "New evidence emerges: Coronavirus 'bioweapon' might have been a Chinese vaccine experiment gone wrong"

	Coronavirus is manmade		The coronavirus is a man-made bioweapon. This narrative does not include articles claiming that the coronavirus was created at the Wuhan Institute of Virology. It rather focuses on articles claiming that the coronavirus was created by the US to target China and Iran, by Russia to target NATO, *etc*.
			E.g. “The [New York] Times admitted [...] that the US created COVID-19 and urgently needs to blame someone else for either using it as an act of war or, more likely, losing control of a biological weapon”
	Link between 5G and coronavirus		There is a link between the deployment of 5G technologies and the spread of the coronavirus.
			E.g. “The coronavirus pandemic [...] actually resulted from the activation of 5G hotspots. Wuhan is a 5G test zone [...] Shortly after activation, the coronavirus was discovered”
	Other conspiracy theories		Any other coronavirus-related conspiracy theories
			E.g. “Pittsburgh Coronavirus Researcher Murdered Close to ‘Very Significant Findings’”
Criticism of the EU and inter/national actors	Criticism of the EU		The coronavirus pandemic is used to criticise the European Union (EU) and sow distrust in its institutions.
			E.g. “The European Union, humiliated by its failure in the face of the coronavirus”
	Criticism of national governments		The coronavirus pandemic is used to criticise and sow distrust in national governments.
			E.g. “Macron's strategy has put us in DANGER! [...] The people must react [...] We must take radical measures to compensate for our [government’s] errors and lack of preparation. As for our ‘leaders,’ we will take care of them once this crisis is over, as they took care of us!”

	Criticism of international organisations	Anti-WHO narratives	The coronavirus pandemic is used to criticise and sow distrust in the World Health Organisation (WHO).
			E.g. "Evidence shows WHO severely overstated the fatality rate of the coronavirus leading to the greatest global panic in history"
		Anti-UN narratives	The coronavirus pandemic is used to criticise and sow distrust in the United Nations (UN).
			E.g. "If we are to believe feminist UN Secretary General António Guterres, this [the coronavirus pandemic] is some kind of primordial conspiracy of the patriarchy"
		Anti-NATO narratives	The coronavirus pandemic is used to criticise and sow distrust in the North Atlantic Treaty Organisation (NATO).
			E.g. "The NATO military alliance is showing a rather unbecoming dog-eat-dog individualistic reaction in coping with the coronavirus pandemic"
Claims of authoritarianism and dystopia	Turn to authoritarianism		The coronavirus pandemic is being used to implement authoritarian means, which citizens would not accept under normal circumstances.
			E.g. "The UK government's new Covid rules are authoritarian and arbitrary. How much longer will people put up with this?"
	Censorship by digital platforms		Digital platforms (Facebook, Twitter, YouTube, *etc*.) censor dissenting voices under the pretext of fighting misinformation.
			E.g. "Twitter, Facebook & Google are pure evil for not even allowing alternative voices on Covid"
	Censorship of dissenting voices		Dissenting voices are being censored through authoritarian means. This narrative does not include articles on the actions of digital platforms. It rather focuses on articles on the actions of the authorities.

			E.g. “Corona fascism - the 'war on free speech and dissent' [...] Rather than engage in reasonable debate about the issues, the government has instead waged war on anybody disagreeing with their ever disintegrating narrative”
	Dystopia		The coronavirus pandemic is being used to implement a dystopic, Orwellian-style surveillance state.
			E.g. “The emerging totalitarian dystopia […] Few phenomena have had an impact around the world as quickly as the current coronavirus outbreak. In a very short time, human life has been completely reorganised”
Geopolitics	Pro-Russia narratives	Vaccine and other drugs	Russia has developed a safe and effective COVID-19 vaccine - Sputnik V - as well as many effective COVID-19 treatments.
			E.g. “Russia officially registered the first corona vaccine, making a global breakthrough. In an interview with RT, a British expert assessed the importance of this event and its contribution to a faster end to the corona pandemic”
		International help	Russia gladly aids countries that are strongly hit by the coronavirus pandemic.
			E.g. “Extending a Helping Hand: How Russia is Aiding Italy's Fight Against Raging COVID-19 Pandemic”
		West is russophobic	The West is russophobic and this prevents it from collaborating with Russia, even when the collaboration would benefit both parties.
			E.g. “Russia criticises France's call not to use [the Russian vaccine,] ‘It is a hybrid form of racism’”
		Management of the pandemic	Russia is managing the pandemic superbly.
			E.g. “WHO: ‘Russia has been one step ahead of the covid-19 epidemic

			thanks to excellent measures taken from day one'"
		Other pro-Russia narratives	Any other pro-Russia narratives
			E.g. "Russia is developing biochips to detect SARS-CoV-2"
	Anti-China narratives	Responsible for pandemic	China is responsible for the pandemic. E.g. China was not transparent about the coronavirus outbreak in Wuhan and is thus responsible for the spread of the virus. This narrative includes articles that refer to the coronavirus as "the Chinese virus."
			E.g. "President Donald Trump came under fire for his use of the phrase "Chinese virus" to describe the coronavirus [...] The virus did indeed originate in China. Most of all, that the Chinese government should be shielded in any way from the results of its own pathological and tyrannical misgovernment is perverse beyond measure"
		China cannot be trusted	China cannot be trusted - Even after the first months of the pandemic, it is not transparent about COVID-19 cases because it wants to appear to have the situation under control or it exaggerated the severity of the coronavirus in order to force the rest of the world into lockdown, *etc*.
			E.g. "The Chinese government launched an aggressive propaganda campaign to exaggerate the severity of coronavirus in order to force the rest of the world into a draconian lockdown that would serve to benefit Beijing"
		Faulty medical equipment	China delivers faulty medical equipment to countries in need, in a self-interested attempt to spread its soft power.
			E.g. "China is flooding Europe with defective medical equipment [...] China is trying to expand its economic power through 'predatory aid offers' [...] China seems to be ignoring

			normal rules of conduct in every area of everyday life”
		Other anti-China narratives	Any other anti-China narratives
			E.g. “China’s Disgusting Profiteering from Coronavirus Spread Exposed”
	Anti-US narratives		The US is mismanaging the pandemic, is responsible for the spread of the coronavirus, *etc*.
			E.g. “The United States is a leader in the spread of coronavirus”
	Pro-China narratives	Vaccines	China has developed safe and effective COVID-19 vaccines, which are being administered globally.
			E.g. “Victor Orban has indicated that he will choose the Chinese preparation because he trusts it the most”
		International help	China is playing a crucial role in aiding countries that are strongly hit by the coronavirus pandemic.
			E.g. “China Is Saving the World From COVID-19 [...] China understands its responsibility to the rest of the world as a global leader, and that’s why it’s dedicated itself to helping everyone else who requests its assistance”
	Anti-West narratives		The West is behaving aggressively towards Russia and China, trying to politicise the vaccines and the origin of the coronavirus, *etc*.
			E.g. “The head of Russian diplomacy returned to the words of Emmanuel Macron, who accused Beijing and Moscow of waging a ‘war’ on vaccines [...] ‘Obviously, there are attempts to politicise the situation’”
	Other geopolitical narratives		Any other geopolitical narratives
			E.g. “The cover-up and lies of the Iranian leaders are some of the underlying reasons for the spread of the coronavirus to other countries”

Health-related narratives	Unconfirmed claims		Unconfirmed or false claims about the origin and spread of the coronavirus, COVID-19, its symptoms, *etc*.
			E.g. “Only 1 per cent of infected coronavirus patients in hospital are women”
	Unconfirmed treatments	Chloroquine	Chloroquine and hydroxychloroquine are effective COVID-19 treatments, but the authorities prefer pushing vaccines, which are more lucrative.
			E.g. “Chloroquine banned in France: it was banned because it does not bring revenue to Big Pharma, and it does not matter if it saves lives!”
		Ivermectin	Ivermectin is an effective COVID-19 treatment, but the authorities prefer pushing vaccines, which are more lucrative.
			E.g. “Ivermectin: Highly effective against Corona, but fought by WHO & mainstream”
		Vitamins	Vitamins - C, D, *etc*. - are effective COVID-19 treatments. They also prevent COVID-19, and are preferable to vaccines.
			E.g. “Give people vitamin D instead of vaccinating them! A [vitamin D] deficiency is the cause of nine out of ten Covid-19 deaths!”
		Remdesivir	Unconfirmed or false claims about the effectiveness of remdesivir for the treatment of COVID-19. While remdesivir has been approved for emergency use to treat COVID-19 in around 50 countries, unverified sources claim that it is “toxic” and even “deadly” for COVID-19 patients. Other unverified sources claim that the approval of remdesivir over other drugs was financially motivated.
			E.g. “Most ‘Covid-19’ Deaths were a direct result of the administration of [...] Remdesivir”

		Other unconfirmed treatments	Any other unconfirmed or false treatments
			E.g. “Hospitalised COVID Patient Makes Miraculous Recovery After Budesonide Treatment — Big Pharma Does Not Want You To See This!”
	Home remedies		“Home remedies” can both prevent coronavirus infection and cure COVID-19. Honey, turmeric tea, a keto diet, artemisia annua or wormwood, *etc*. prevent coronavirus infection. Hot whisky with honey, mursal tea, black cumin, *etc*. cure COVID-19.
			E.g. “Coronavirus cure: It's so simple: Whiskey and honey!”
Fearmongering	In general		Fearmongering reporting on the coronavirus pandemic
			E.g. “Coronageddon 2: FEMA Orders 100,000 More Body Bags”
	New wave(s)		Fearmongering reporting on new waves of the pandemic
			E.g. “USA says a Fourth Wave is coming and ‘the Vaccine is Not Perfect,’ therefore you still have No Rights”
	New variant(s)		Fearmongering reporting on new variants of the coronavirus pandemic
			E.g. “Nightmarish discovery of a COVID-19 mutant from South Africa”
	Other diseases		Fearmongering reporting on new diseases that might follow the coronavirus pandemic
			E.g. “Report: Different ‘Pandemic Potential’ Brain Destroying Virus With 75 per cent Death Rate Spreading in India”
Downplaying COVID-19	Coronavirus hysteria		There is a general state of hysteria over the coronavirus pandemic that portrays COVID-19 as much more dangerous than it is.
			E.g. “We don't have a coronavirus pandemic, but a pandemic of fear”

	COVID-19 cases and/or deaths overestimated		COVID-19 cases and deaths are overestimated. Flu patients are counted as COVID-19 patients. All those who die while positive for the coronavirus are counted as COVID-19 deaths, regardless of their cause of death.
			E.g. “40 per cent Of Colorado County’s COVID Deaths Were Actually Gunshot Wounds [...] Authorities were counting any dead people who tested positive for coronavirus in the last 30 days as ‘deaths among cases’”
	Tests are unreliable		COVID-19 tests are unreliable, they produce false positives, leading to higher counts of “COVID-19 cases” than there actually are.
			E.g. “Closer analysis shows that PCR tests are actually quite blotchy and there are many false positives [...] ‘A large number of people who may be carrying relatively insignificant amounts of the virus’ are considered infected. However, the severity of the infection is never quantified, which is essentially a false positive. Your level of infection is essentially zero.”
	COVID-19 not more dangerous than flu		COVID-19 is not more dangerous than the flu. Just like the flu, it is not lethal for healthy people. Its mortality rate is similar - if not even lower - than that of the flu.
			E.g. “Mortality 2020 vs 2017: ‘Covid-19 is actually less fatal than the flu’”
	Pandemic is (almost) over		The pandemic is over, and we can go back to normal life, or the pandemic is almost over, and we will be able to go back to normal life soon.
			E.g. “The spread of the coronavirus in the population has stopped. Life can begin to return to normal”
Anti-minority narratives	Anti-immigrant narratives		Immigrants are super spreaders of the coronavirus. They do not have to or refuse to comply with coronavirus restrictions.

			E.g. “Coronavirus, Rome residents revolt: ‘We are in quarantine while migrants are out for a walk”
	Anti-Muslim narratives		Muslims are super spreaders of the coronavirus. They do not have to or refuse to comply with coronavirus restrictions. They also celebrate the “infidels” dying of COVID-19.
			E.g. “As we all know, some Muslims haven't really gone to great lengths to comply with containment measures, masks and restrictions on gatherings”
	Other anti-minority narratives		Any other anti-minority (antiziganist, anti-Semitic, *etc*.) narratives
			E.g. “Outrageous! Gypsies dance in a hospital while patients wait for hours to be admitted”
Distrust towards media	Media		Mainstream media cannot be trusted, they are an instrument of propaganda.
			E.g. “The mainstream media [...] would have us believe that 15,503 people have died from COVID-19 in the last year. But this turns out to be pure manipulation”
	Fact checkers		Fact checkers cannot be trusted, they have their own economic interests, and they act as a “ministry of truth,” suppressing alternative voices.
			E.g. “‘Fact checkers were established to contrast freedom of expression and diversity of opinion”
Other	Sensational anecdotes		Clickbait, sensational reporting
			E.g. “Woman Arrested After Allegedly Licking Nearly $2,000 Worth of Items at Grocery Store”
	VIPs with COVID-19		Reports of VIPs with COVID-19
			E.g., “Tom Hanks and his wife recover from coronavirus in Australia”
	Criticism of Fauci		Criticism of Dr Anthony Fauci, the US government’s chief medical adviser

			E.g. “Dr Anthony Fauci has been the leader of the federal government’s politically based responses to Covid-19 [...] But – keep your fingers crossed – that may be about to change. The serious unravelling of Fauci’s undeserved reputation as ‘an American hero’ and ‘the most trusted voice on Covid-19’ has finally begun”
	Anti-Greta Thunberg narratives		Climate activist Greta Thunberg is criticised for trying to shift the world’s attention from the coronavirus pandemic to climate change, for speaking about the coronavirus pandemic as if she were a health expert, *etc*.
			E.g. “Greta Thunberg is suffering from a lack of attention these days [...]. She initially wanted to gain attention by claiming that she may be infected with covid-19, but when that didn't work out, she resumed insisting that [...] global warming still needs to be fought”
