# Supplementary material for: Trend analysis of COVID-19 mis/disinformation narratives–A 3-year study
Source: PLoS One. 2023 Nov 17;18(11):e0291423. doi: 10.1371/journal.pone.0291423 (PMC10655972; doi:10.1371/journal.pone.0291423)
Supplement: S1 Table — The table shows precision, recall and F1 score for the supernarratives. (PDF) [file pone.0291423.s003.pdf]

Supernarrative	Precision	Recall	F1-score
**Anti-minority narratives**	0.83	0.84	0.83
**Claims of authoritarianism and dystopia**	0.82	0.79	0.80
**Conspiracy theories**	0.83	0.82	0.83
**Criticism of EU and inter/national actors**	0.77	0.70	0.74
**Criticism of restrictions**	0.82	0.82	0.82
**Distrust towards media**	0.85	0.73	0.79
**Downplaying COVID**	0.79	0.70	0.74
**Fearmongering**	0.73	0.83	0.78
**Geopolitics**	0.85	0.83	0.84
**Health-related narratives**	0.78	0.82	0.80
**Not-Apply**	0.84	0.89	0.86
**Vaccine-related narratives**	0.92	0.90	0.91
**Other**	0.77	0.69	0.73
