# Supplementary figures and images for: Trend analysis of COVID-19 mis/disinformation narratives–A 3-year study
Source: PLoS One. 2023 Nov 17;18(11):e0291423. doi: 10.1371/journal.pone.0291423 (PMC10655972; doi:10.1371/journal.pone.0291423)

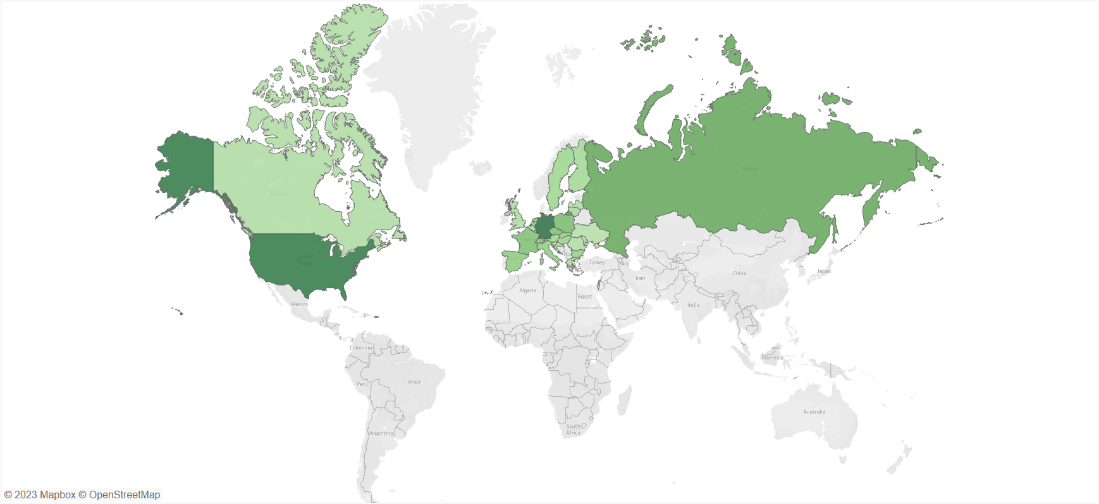

Supplement: S1 Fig — The map illustrates the country coverage of our database of 460 unverified sources, clearly showing that the databased of unverified sources this investigation is based on contains many US, Western European, and Russian sources while it is lacking Latin American, African, and Asian sources. (TIF) [file pone.0291423.s004.tif]

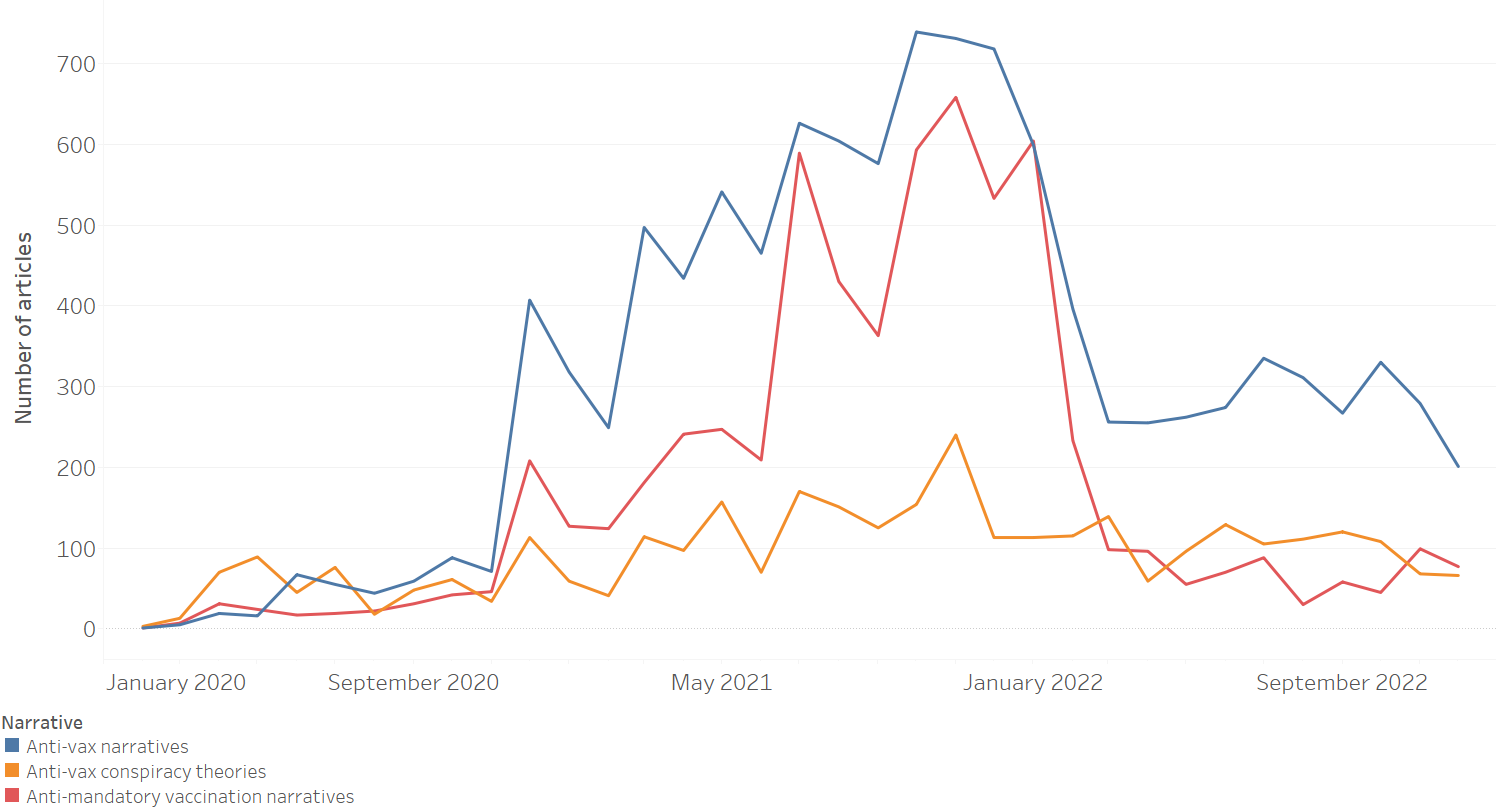

Supplement: S2 Fig — The timeline shows the spread over time of “anti-vax narratives,” “anti-mandatory vaccination narratives,” and “anti-vax conspiracy theories”. (TIF) [file pone.0291423.s005.tif]

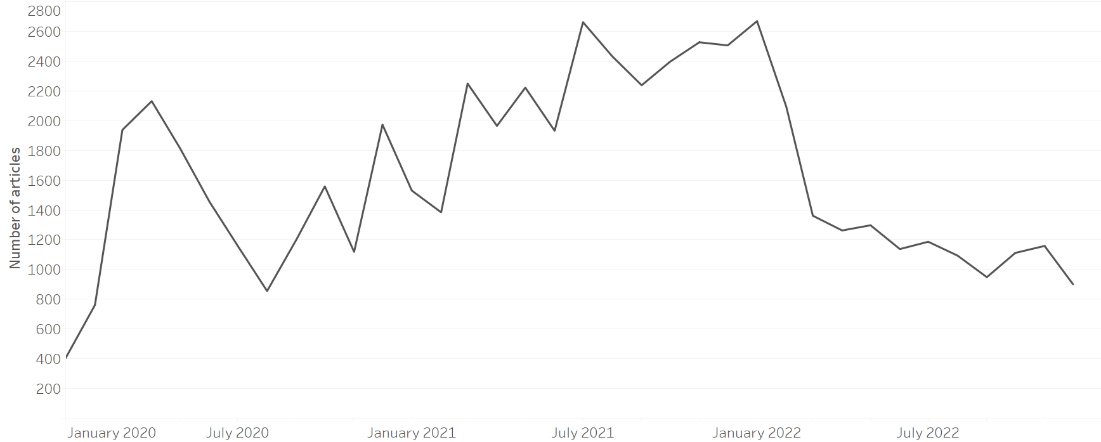

Supplement: S3 Fig — The timeline shows the overall distribution of all annotated COVID-19 related data. (TIF) [file pone.0291423.s006.tif]
