# Supplementary material for: Trend analysis of COVID-19 mis/disinformation narratives–A 3-year study
Source: PLoS One. 2023 Nov 17;18(11):e0291423. doi: 10.1371/journal.pone.0291423 (PMC10655972; doi:10.1371/journal.pone.0291423)
Supplement: S1 File — (PDF) [file pone.0291423.s001.pdf]

-{!babel m=m t="beer el-hadjadj merscorp mittelerde+rollenspie per cent mortgage+electronic+registration+system per cent municipal+employee per cent"} +({!babel m=m t="2019-n-CoV 2019nCov 2019冠状病毒病 omicron5 B+1+1+529 bliskoistočni+respiratorni+sindrom corona+virüs per cent corona-virus coronaviren coronavirus coronavirus+mers coronavirus-fälle coronavirus-infektionen coronaviruses coronavirussen coronavírus coronavírus+mers COVID covid-19 covid-19-Pandemie covid-pandemie covid19 korona+virus korona+virusas koronavirus koronavīruss koronawirus per cent koroona-viirusnakkus koroonawir per cent mers+coron per cent mers+cov mers-coronavirus mers-cov middle+east+respiratory+syndrom per cent middle+east-respiratory+syndrom per cent même+famille+du+sras now+koronawirus per cent nuevo+virus+como+el+sars Omicron Omikron Omikron-varianten omikronvariant orta+doğu+solunum+sendromu per cent ressemblant+au+sras ressemble+au+syndrome+respiratoire+aigu+sévère same+family+as+sars SARS+corona sars+lignende sars+like sars+liknande sars+ähnlich per cent SARS-CoV sars-cov-2 SARS-CoV-2-Omikron SARS-CoV-2-omikronvariant sars-lignende sars-like sars-liknande sars-ähnlich per cent semblable+au+sras similar+to+sars simile+alla+sars stessa+famiglia+della+sars síndrome+respiratorio+de+oriente+medio varian+Omicron variante+ómicron vi+rút+corona virus+corona virus+from+sars+family wirus per cent+corona Ômicron κοροναϊ per cent κοροναϊός κορωναϊός μετάλλαξη+Όμικρον Μετάλλαξη+Όμικρον корона+вирус коронавирус per cent Коронавирустар Омікрон-варіант Омикрон-штамм נגיף+הקורונה נגיף+קורונה קורונה הקורונה+–וירוס الكورنوفايروس حمة+الكورونا فيروس+ا+كيل فيروس+تاجي فيروس+كوروني فيروسات+إكليلية فيروسة+مكللة كرونا+ويروس كروناويروس كورونا+ويروس كوروناويروس ក+រ+ណ+ប+រភ+ទថ+ម ក+រ+ណ+វ+រ+ស ក+រ+ណ+វ+រ+សប+រភ+ទថ+ម ជ+ង+រល+កស+ត ម+រ+គក+រ+ណ ម+រ+គក+រ+ណ+វ+រ+ស វ+រ+សក+រ+ណ კორონა+ვირუსი ომიკრონ+ვარიანტ per cent コロナウイルス 严重特殊传染性肺炎 冠状病毒 冠狀病毒 新冠狀病毒 新冠病毒 新冠肺炎 新型冠状病毒肺炎 新型冠狀病毒感染 武汉肺炎 起2019新型冠狀病毒 중동호흡기증후군+코로나바이러스 코로나바이러스"} (+{!babel m=m t="Centaurus BA+2+75"} +{!babel m=m t="virus per cent coronavirus per cent covid19 covid-19 épidémie subvariant per cent allarm per cent mutazione sintom_ pandemic sotto-variante virolog_ covid per cent variante variant sottovariante pandemia omicron virologist per cent subvariant omicron5 síntomas Ómicron"} -{!babel m=m t="arcigay"}) (+{!babel m=m t="ក+រ+ណ+វ+រ+ស ម+រ+គក+រ+ណ+វ+រ+ស ក+រ+ណ+ប+រភ+ទថ+ម វ+រ+សក+រ+ណ ក+រ+ណ+វ+រ+សប+រភ+ទថ+ម"} +{!babel m=m t="ករណ ក+នឡ+ង ស+ល+ប បង+ករអ+យស+ល+ប មន+ទ+រព+ទ+យ ស+ថ+តន+ក+ន+ងក+រត+មជ+ន ផ+ល+វជង+ហ+ម ស+ថ+នភ+ពធ+ងន+ធ+ងរ គ+រអ+យកត+ស+គ+ល"} -{!babel m=m t="ក+រប+រគ+តន+ត+រ ច+រ+ង វង+តន+ត+រ រ+ង រ+ងភ+គ ព+ណ+ជ+ជកម+ម"}) (+{!babel m=m t="patient patiente patients patientes mort morte morts mortes mourir mortel mortelle mortels mortelles décès décédé décédée décédés décédées décéder cas hôpital hôpitaux hospitalisé hospitalisée hospitalisés hospitalisées Rassemblement Rassemblements épidémie épidémies flambée suspect suspects confirmé confirmés virus"} +{!babel m=m t="coronavirus corona+virus per cent"}) (+{!babel m=m t="coronavirus novel+coronavirus corona+virus per cent"} +{!babel m=m t="case cases outbreak outbreaks death deaths epidemic hospital hospitals cluster clusters fatal patient patients confirmed suspected infection virus"}) (+{!babel m=m t="+ {"فيروس فيروسات{!babel m=m t="كورونا الكورونا"}))
